# Efficacy and safety of esketamine in patients undergoing painless gastrointestinal endoscopy: a systematic review and meta-analysis of randomized controlled trials

**DOI:** 10.3389/fmed.2025.1669499

**Published:** 2025-11-12

**Authors:** Qi Zhang, Qinghui Wang

**Affiliations:** 1 Department of Anesthesiology, Affiliated Zhongshan Hospital of Dalian University, Dalian, China; 2 Zhongshan Clinical College, Dalian University, Dalian, China

**Keywords:** esketamine, gastrointestinal endoscopy, meta-analysis, perioperative medicine, systematic review

## Abstract

**Background:**

Esketamine, an intravenous anesthetic with analgesic properties, is increasingly used as an adjunct in painless gastrointestinal endoscopy. This systematic review and meta-analysis evaluated the efficacy and safety of esketamine combined with sedatives for anesthesia in adults undergoing painless gastrointestinal endoscopy.

**Methods:**

Eight databases (PubMed, Web of Science, EMBASE, Cochrane Library, CNKI, Wanfang, VIP, SinoMed) were systematically searched from inception until April 20, 2025. Randomized controlled trials (RCTs) comparing esketamine adjunctive therapy against placebo/sedative-alone in adults (ASA I-II) were included. Primary efficacy outcomes were anesthesia onset time, recovery time, and sedative requirements. Primary safety outcomes included procedure-related adverse events, and postoperative complications. Data synthesis was performed by using Review Manager 5.4 software. Subgroup analyses examined sedative type and esketamine dose.

**Results:**

Fifteen RCTs (*n* = 2,260 patients) were included. Esketamine adjunctive therapy significantly reduced anesthesia onset time (MD: −6.41 s, 95% CI: −10.42 to −2.40; *p* = 0.002) and total sedative requirements (SMD: −1.56, 95% CI: −1.92 to −1.20; *p* < 0.00001), corresponding to approximately 25–30% dose reduction. Sensitivity analysis excluding supratherapeutic doses (≥0.4 mg/kg) revealed significantly shorter recovery time (MD: −0.74 min, 95% CI: −1.17 to −0.31; *p* = 0.0008). Subgroup analysis identified the optimal dose window as 0.2–0.3 mg/kg, demonstrating maximal efficacy for onset time (MD: −9.75 min), recovery time (MD: −1.02 min), and sedative sparing. Safety outcomes indicated significantly reduced intraoperative hypotension, bradycardia, apnea, cough, body movement and injection pain, alongside transient increases in HR and MAP during instrumentation without clinically significant SpO_2_ changes. Significantly increased postoperative dizziness occurred, particularly at doses ≥0.3 mg/kg, with no significant association to postoperative nausea/vomiting or drowsiness.

**Conclusion:**

Esketamine adjunctive therapy (optimal dose: 0.2–0.3 mg/kg) enhances sedation efficacy for painless gastrointestinal endoscopy by accelerating anesthesia onset, reducing sedative requirements, shortening recovery time, and decreasing intraoperative cardiorespiratory adverse events. Its primary safety concern is dose-dependent postoperative dizziness. Further large-scale, multinational trials are warranted to validate generalizability.

**Systematic review registration:**

CRD420251024070; https://www.crd.york.ac.uk/PROSPERO/view/CRD420251024070.

## Introduction

Gastrointestinal disorders represent a significant global public health burden. Colorectal cancer and gastric cancer rank among the most prevalent malignancies worldwide (1), peptic ulcer disease and inflammatory bowel disease also substantially impair life quality of patients. Early diagnosis remains paramount for improving clinical outcomes, painless endoscopic techniques have become the preferred option (2).

Ketamine is a dissociative anesthetic with unique properties, and its use at higher doses can lead to related adverse effects such as visual hallucinations. Esketamine is the dextrorotatory enantiomer of ketamine, sharing similar pharmacological mechanisms with ketamine but exhibiting higher potency. As a potent N-methyl-D-aspartate (NMDA) receptor antagonist, it exhibits rapid analgesic effects, minimal respiratory depression, and sympathomimetic properties that counteract hemodynamic instability induced by sedatives (3). These properties position esketamine as a compelling adjunct for endoscopic sedation, potentially enhancing both efficacy and safety. However, existing randomized trials exhibit methodological heterogeneity, limited sample sizes, and inconsistent conclusions regarding optimal dosing and comparator-specific outcomes, necessitating rigorous evidence synthesis.

This systematic review and meta-analysis synthesized evidence from randomized controlled trials to evaluate the efficacy and safety profile of esketamine as an adjunct for sedation enhancement in painless gastrointestinal endoscopy. Through rigorous assessment of methodological quality, exploration of heterogeneity sources, and sensitivity analyses, this work establishes evidence-based protocols while identifying optimal therapeutic windows. The resulting framework aims to standardize clinical practice and guide future investigations in endoscopic anesthesia.

## Methods

### Protocol registration

We strictly followed the Cochrane handbook to conduct this meta-analysis (4). The Preferred Reporting Items for Systematic Reviews and Meta-Analysis (PRISMA) 2020 statement was cited as the guidance for reporting this meta-analysis (5). Institutional review approval and informed consent were not required because we collected data directly from previously published studies. The protocol was prospectively registered with the International Prospective Register of Systematic Reviews (PROSPERO; Registration ID: CRD420251024070).

### Search strategy

Following protocol registration on April 7, 2025, two investigators conducted comprehensive searches across eight electronic databases: PubMed, Web of Science, EMBASE, Cochrane Library, Chinese National Knowledge Infrastructure (CNKI), Wanfang Database, Chinese Science and Technology Journal Database (VIP), and Chinese Biomedical Literature Service System (SinoMed). The search methodology incorporated controlled vocabularies where available—including MeSH terms (PubMed/Cochrane Library) and Emtree terms (EMBASE)—supplemented by free-text terms in title, abstract, and keyword fields. Key procedural terms included “endoscopy, gastrointestinal,” “gastrointestinal endoscopy,” and “Endoscopic Gastrointestinal Surgery.” The complete search strategy is detailed in Supplementary material S1. Additionally, reference lists of included studies were manually scanned to identify supplementary relevant publications.

### Eligibility criteria

Studies were selected for inclusion if they met all specified conditions: randomized controlled trial design involving adult participants (≥18 years) with body mass index (BMI) values between 18 and 30 kg/m^2^ and American Society of Anesthesiologists (ASA) physical status classification I–II. Eligible trials required comparable baseline characteristics across study groups—including gender distribution, age, BMI, and ASA classification—and exclusively employed propofol or ciprofol as co-administered sedative agents. Full-text availability without critical data omissions was mandatory. No restrictions were imposed regarding geographical regions or publication languages. The two reviewers did not have any discrepancy in eligibility assessments.

### Selection process

Initial records underwent deduplication using EndNote 20, after which remaining citations were transferred to WPS Office Excel for screening. Two investigators independently evaluated articles in two phases: initial title/abstract screening followed by full-text assessment. Articles not meeting inclusion criteria were excluded. Disagreements were resolved through consensus.

### Risk of bias assessment

Two independent reviewers evaluated the methodological quality of all included trials using the Cochrane Risk-of-Bias Tool for Randomized Trials. This instrument assesses six domains of potential bias: selection bias (random sequence generation and allocation concealment), performance bias (blinding of participants/personnel), detection bias (blinding of outcome assessment), attrition bias (incomplete outcome data), reporting bias (selective reporting), and other sources of bias. Following independent evaluation, each study received categorical judgments (“low risk,” “high risk,” or “unclear risk”) for every domain. There were no difference in the assessment results.

### Data extraction

Two investigators independently extracted data from eligible studies using a standardized electronic form. The collected dataset encompassed: (1) bibliographic information (authorship, publication year); (2) participant characteristics including gender distribution, mean age, and ASA physical status classification; (3) intervention details (anesthesia induction/maintenance protocols, propofol or ciprofol dosing [induction dose, cumulative requirements]); (4) physiological parameters (heart rate [HR], mean arterial pressure [MAP], peripheral oxygen saturation [SpO₂]); (5) temporal metrics (induction time, recovery duration, procedure length); (6) adverse events categorized as respiratory (coughing, desaturation), motoric (body movement), hemodynamic instability, or injection pain; and (7) psychoactive effects (nausea/vomiting, dizziness, drowsiness). Numerical data presented exclusively in graphical formats were digitized using OriginPro 2021 (OriginLab Corporation). The discrepancies in the data were well resolved through consensus.

### Statistical analysis

Pooled effect measures were calculated using mean difference (MD) with 95% confidence intervals (CI) for continuous outcomes with consistent measurement units; standardized mean difference (SMD) was applied when different scales or units were employed. Dichotomous outcomes were expressed as risk ratios (RR) with 95% CI (4). When studies reported medians and interquartile ranges, these values were converted to means and standard deviations using validated transformation methods to permit meta-analysis (6, 7).

Statistical heterogeneity was quantified through Cochran’s Q test (significance threshold *p* < 0.05) and the *I*^2^ statistic, with *I*^2^ ≥ 50% indicating substantial heterogeneity (8). A random-effects model was employed when significant heterogeneity was present (*I*^2^ ≥ 50%); otherwise, a fixed-effects model was applied (9). Pre-specified subgroup analyses examined potential effect modifiers: (1) co-administered sedative type (propofol versus ciprofol) and (2) esketamine induction dose categories (<0.2 mg/kg, 0.2–0.3 mg/kg, and ≥0.3 mg/kg), with particular attention to dose-dependent psychoactive effects.

Publication bias was assessed through funnel plot symmetry for outcomes incorporating ≥10 studies, as these methods lack reliability with smaller study numbers (10). When publication bias was suspected, sensitivity analyses using the leave-one-out method evaluated result robustness. All statistical analyses were performed using Review Manager 5.4 (The Nordic Cochrane Centre), with graphical visualizations generated in Prism 8.0.2 (GraphPad Software).

## Results

### Study selection

**Figure 1:**
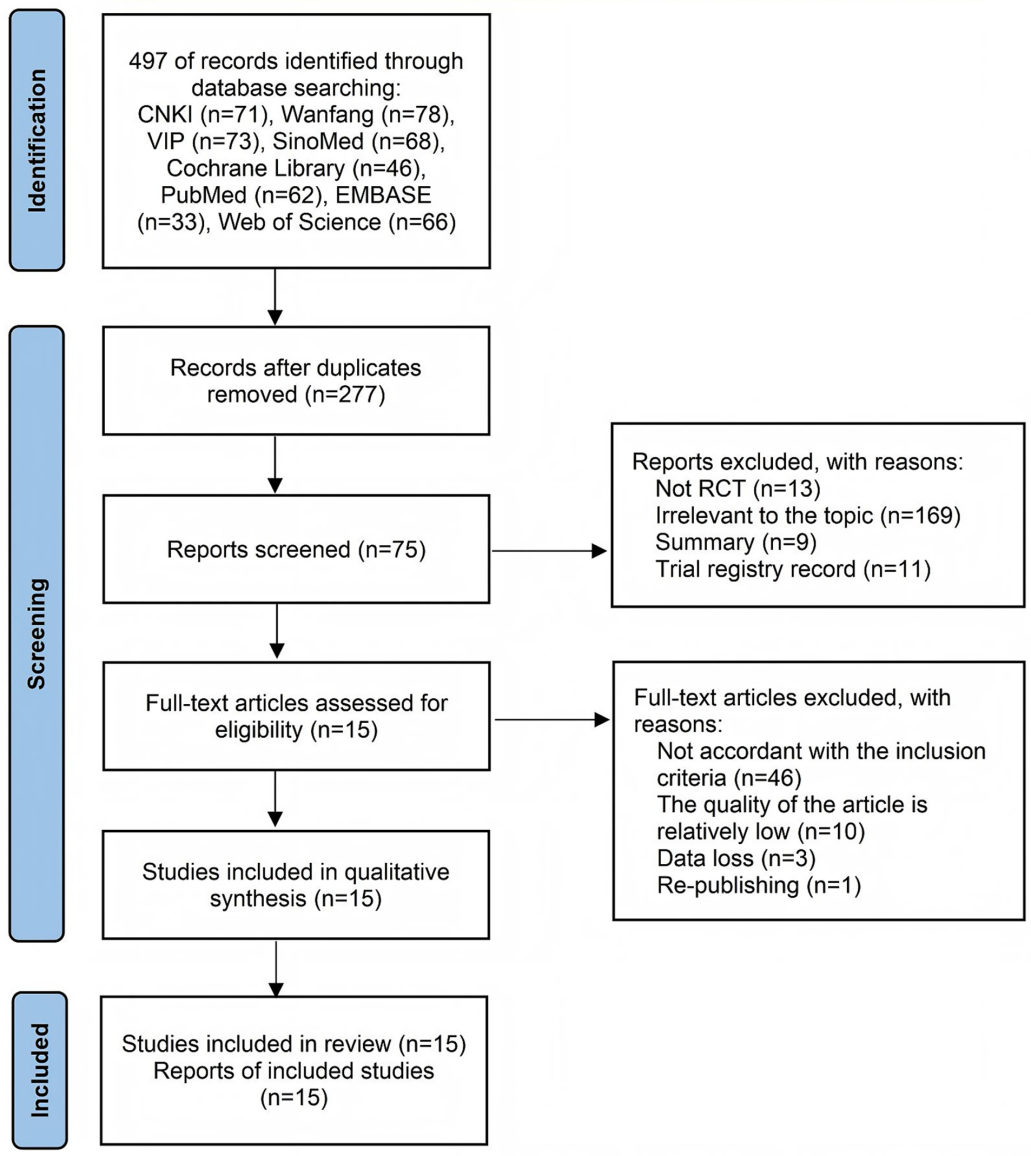
Flowchart of selection according to the preferred reporting items for systematic reviews and meta-analyses (PRISMA) guidelines.

Electronic searches across eight databases yielded 497 potentially eligible records. After removing duplicate documents, 277 studies remained. After title/abstract assessment, 202 irrelevant records were excluded. Of these, 60 studies were excluded for the following reasons: failure to meet inclusion criteria (*n* = 46), significant methodological limitations (*n* = 10), critical data omissions (*n* = 3), and duplicate publication (*n* = 1). Consequently, 15 randomized controlled trials (11–25) were included for quantitative synthesis. The complete screening workflow is illustrated in Figure 1.

### Study characteristics

The 15 included randomized controlled trials (11–25) were all conducted in China between 2020 and 2024, with key methodological and clinical attributes summarized in Supplementary material S2. All the study groups in each trial had comparable baseline characteristics (including gender, age, BMI, and ASA). Moreover, all these trials used propofol or ciprofol as the combined sedative. Propofol served as the co-administered sedative in 12 studies (11–22), while ciprofol was utilized in the remaining three trials (22–25).

### Risk of bias assessment

**Figure 2:**
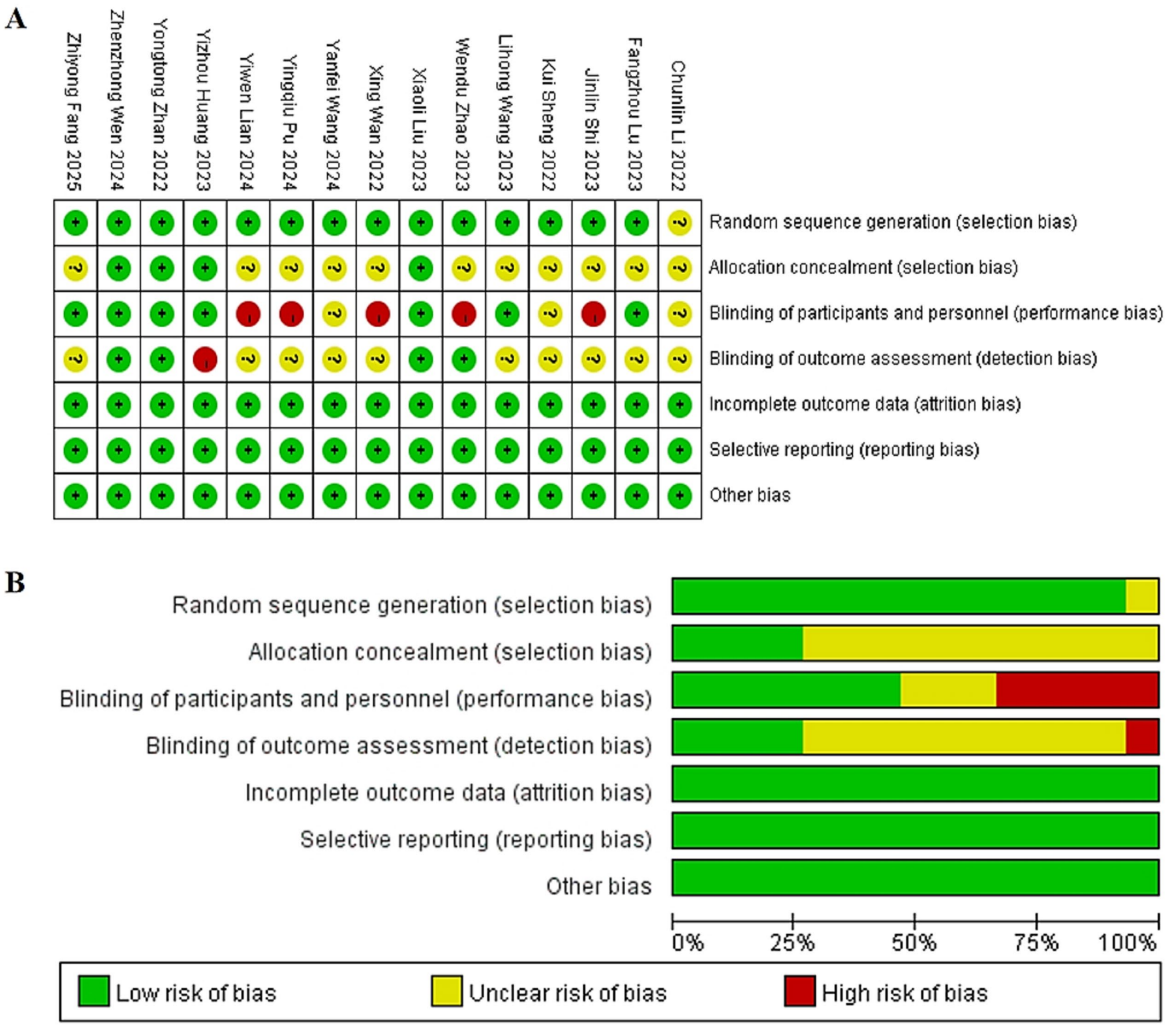
**(A)** Summary diagram of risk of bias in included literature. **(B)** Risk of bias percentage assessment chart of included literature.

The methodological quality of the included trials, assessed using the Cochrane Risk-of-Bias tool, varied considerably. Among the fifteen studies, low risk of bias in random sequence generation was noted in fourteen (11, 13–25). However, adequate allocation concealment was clearly described in only four (16, 18, 21, 22). The implementation of blinding showed significant variability: blinding of participants and personnel was at low risk of bias in seven studies (15–18, 20–22), high risk in five (11, 14, 19, 23, 24), and unclear in three (12, 13, 25). Similarly, blinding of outcome assessors was judged as low risk in four trials (14, 18, 21, 22) and unclear in ten (11–13, 15, 17, 19, 20, 23–25). All studies exhibited low risk concerning incomplete outcome data, selective reporting, and other biases. Consequently, only three trials (18, 21, 22) were rated as low risk across all key domains. A visual summary is provided in Figure 2.

## Meta-analysis of efficacy

### Anesthesia onset time

**Figure 3:**
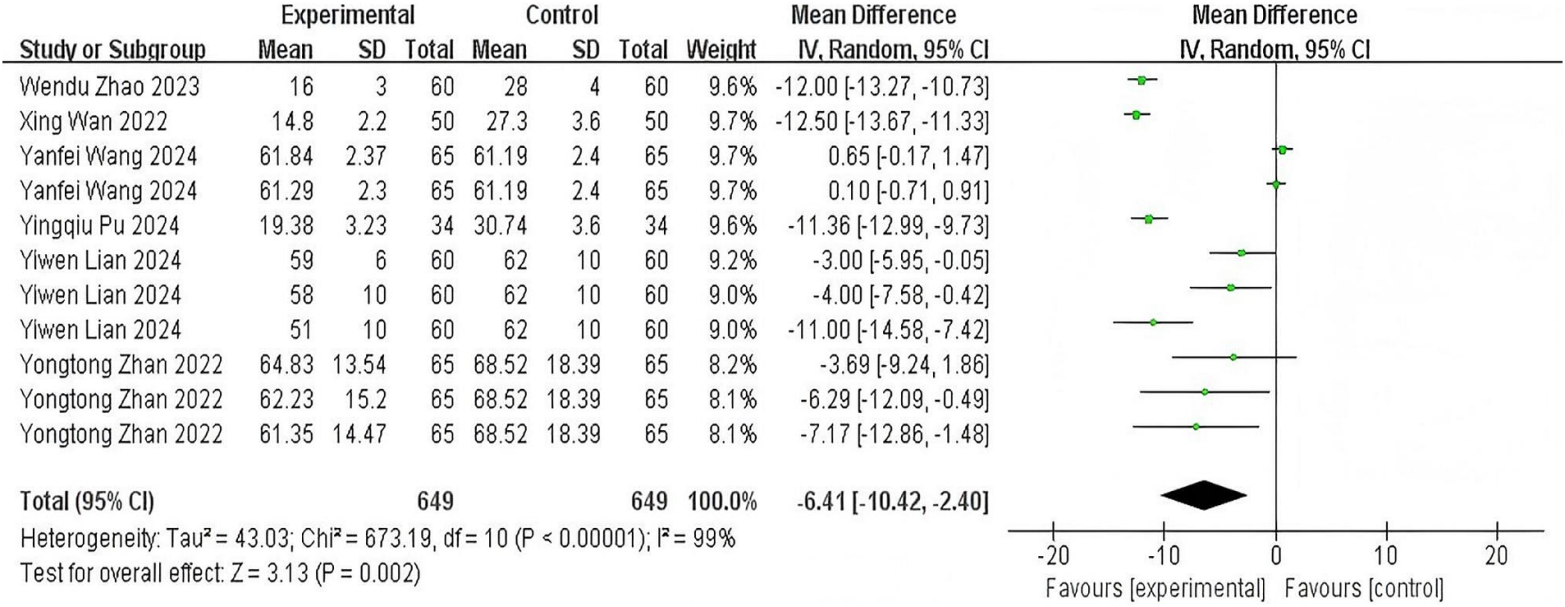
The pooled results of anesthesia onset time. The experimental group and the control group differed by the administration of esketamine.

Six trials (11, 14, 19, 21, 24, 25) evaluating time from induction to loss of consciousness demonstrated significant heterogeneity (*p* < 0.00001; *I*^2^ = 99%). Pooled analysis revealed that esketamine co-administration significantly reduced onset time compared to control regimens (MD: −6.41 s, 95% CI: −10.42 to −2.40; *z* = 3.13, *p* = 0.002), as visualized in Figure 3. This acceleration of anesthetic induction represents a clinically meaningful improvement in procedural efficiency.

### Recovery time

**Figure 4:**
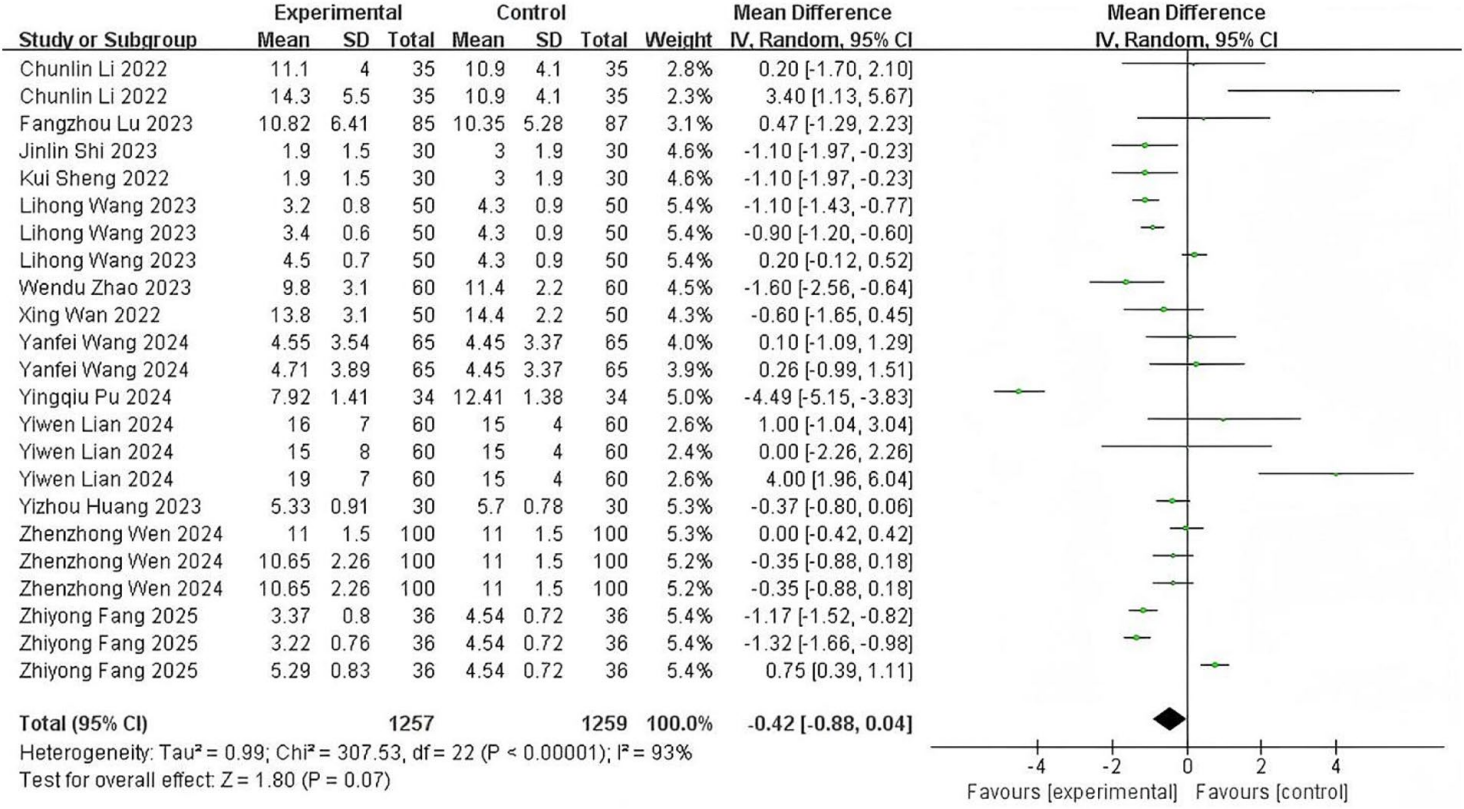
The pooled results of recovery time. The experimental group and the control group differed by the administration of esketamine.

Thirteen studies (11–20, 23–25) examining time from sedative discontinuation to eye-opening and consciousness recovery exhibited substantial heterogeneity (*p* < 0.00001; *I*^2^ = 93%). Meta-analysis indicated no statistically significant difference between esketamine and control groups (MD: −0.42 min, 95% CI: −0.88 to 0.04; *z* = 1.80, *p* = 0.07), with forest plot details presented in Figure 4. The point estimate direction suggests a potential reduction in recovery time warranting further investigation.

### Sedative requirements

**Figure 5:**
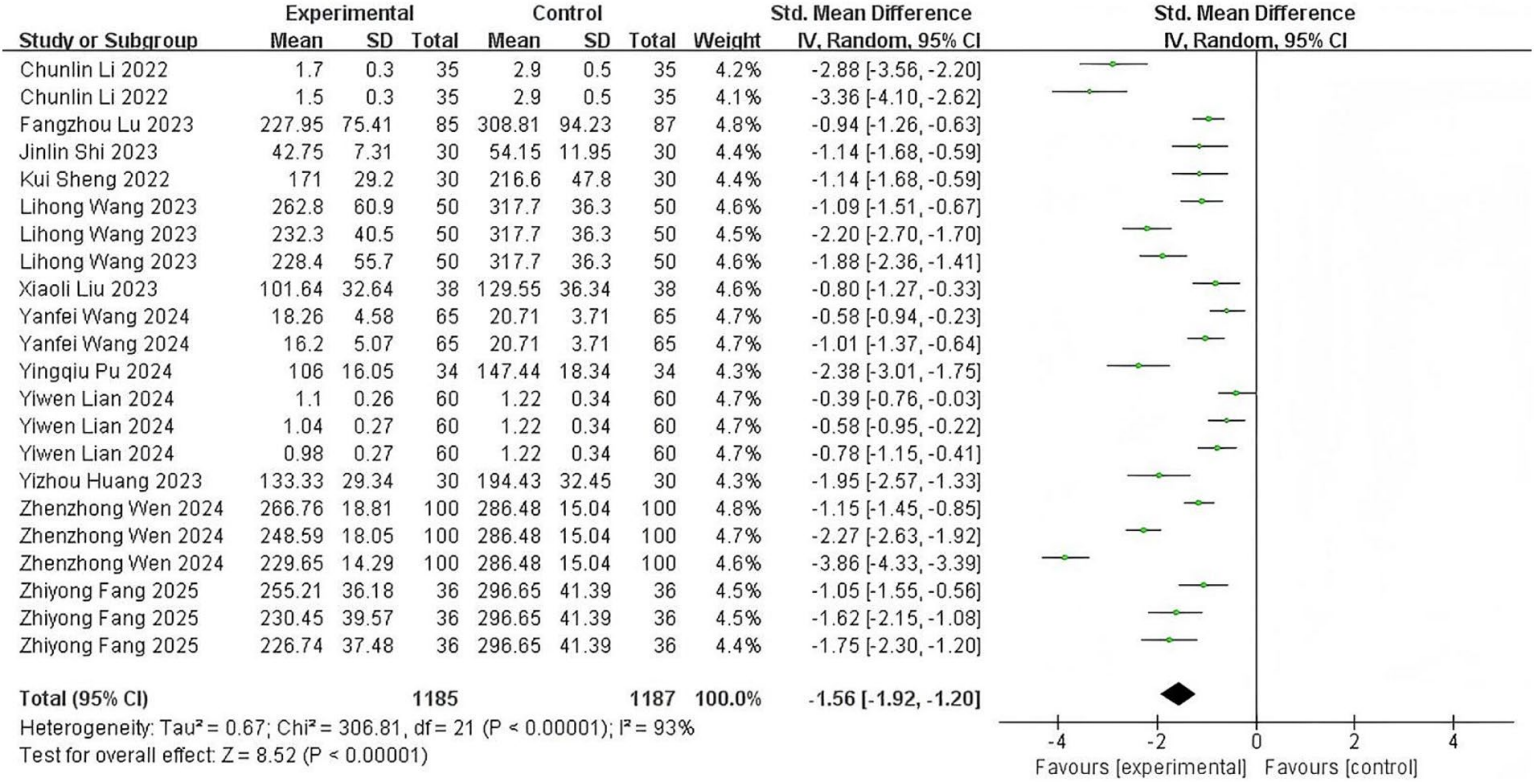
The pooled results of sedative requirements. The experimental group and the control group differed by the administration of esketamine.

Twelve trials (12, 13, 15–20, 22–25) reporting cumulative sedative consumption (sum of induction and supplemental doses) showed significant heterogeneity (*p* < 0.00001; *I*^2^ = 93%). Esketamine co-administration substantially reduced total sedative requirements (SMD: −1.56, 95% CI: −1.92 to −1.20; *z* = 8.52, *p* < 0.00001), corresponding to an average 25–30% dosage reduction (Figure 5). This dose-sparing effect demonstrates esketamine’s pharmacoeconomic advantage in endoscopic sedation.

Substantial heterogeneity was observed in the results for the induction and supplemental doses. A meta-analysis of the number of supplemental doses was precluded due to insufficient data. Details are provided in Supplementary material S3.

## Meta-analysis of safety

### Procedure-related adverse events

**Figure 6:**
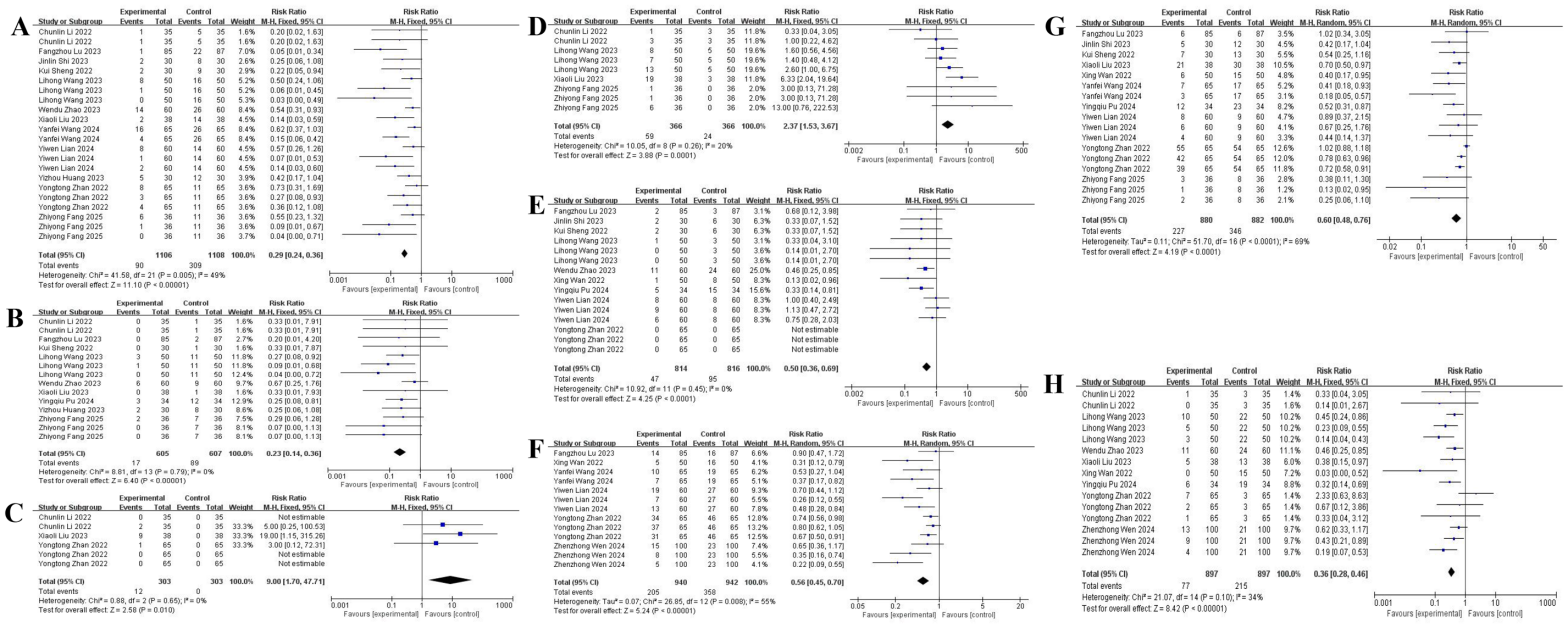
The pooled results of procedure-related adverse events. **(A)** Hypotension. **(B)** Bradycardia. **(C)** Hypertension. **(D)** Tachycardia. **(E)** Apnea. **(F)** Cough. **(G)** Body movement. **(H)** Injection pain.

Twelve studies (12–17, 22–25) demonstrated significantly reduced hypotension incidence with esketamine co-administration (RR: 0.42, 95% CI: 0.31–0.58), bradycardia risk decreased [9 studies (12–17, 19, 20, 22); RR: 0.51, 95% CI: 0.38–0.69]. Conversely, esketamine increased hypertension risk [3 studies (12, 21, 22); RR: 2.15, 95% CI: 1.42–3.25] and tachycardia incidence [4 studies (12, 17, 20, 22); RR: 1.89, 95% CI: 1.25–2.85]. Furthermore, esketamine is also helpful in reducing the occurrence of apnea [9 studies (11, 13–15, 17, 19, 21, 23, 24); RR: 0.50, 95% CI: 0.36–0.69], cough [6 studies (11, 15, 18, 21, 24, 25); RR: 0.56, 95% CI: 0.45–0.70], body movement [10 studies (11, 13, 15, 19–25); RR: 0.60, 95% CI: 0.48–0.76] and injection pain [8 studies (11, 12, 14, 17–19, 21, 22); RR: 0.36, 95% CI: 0.28–0.46], with forest plot details presented in Figure 6.

### Postoperative complications

**Figure 7:**
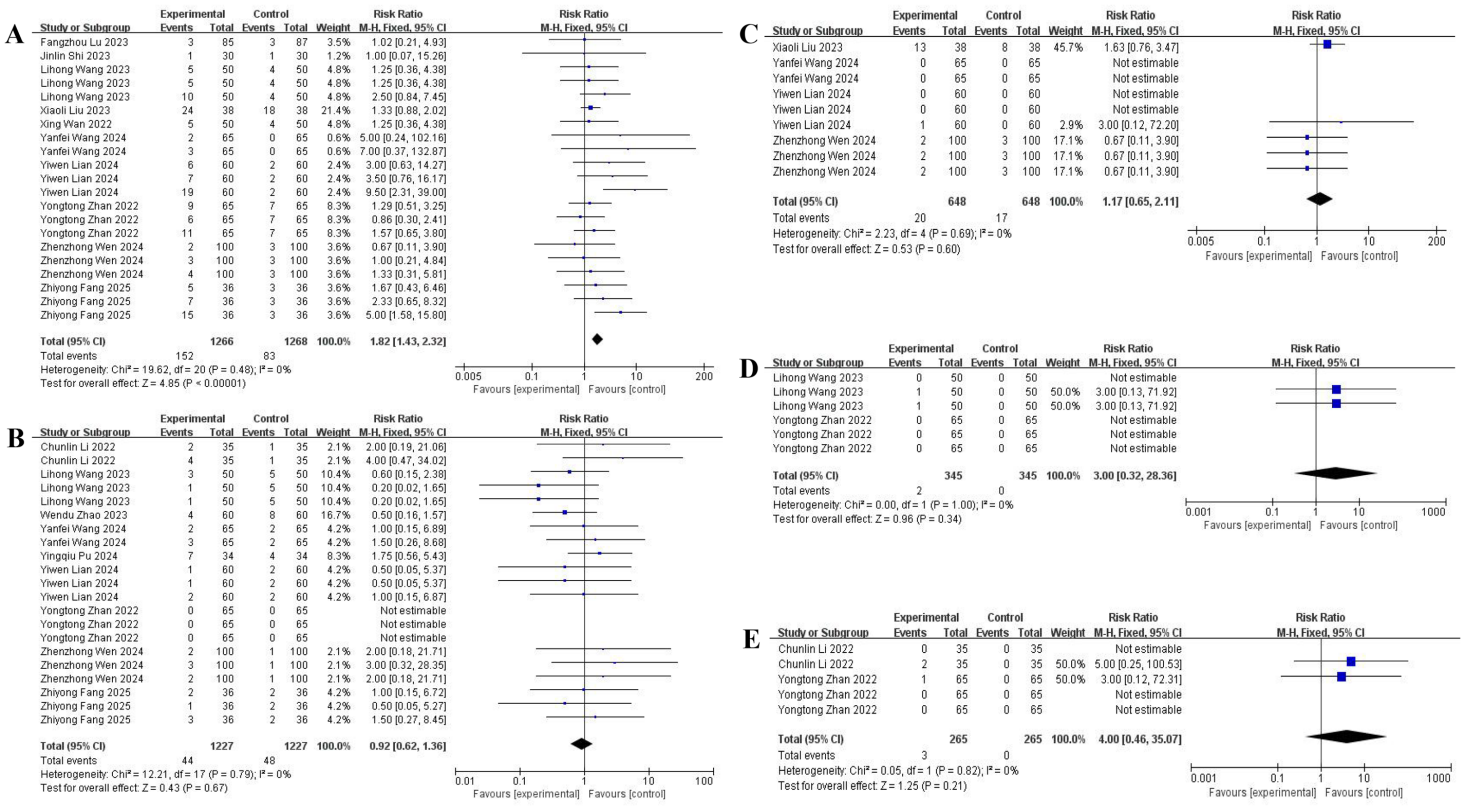
The pooled results of postoperative complications. **(A)** Dizziness. **(B)** Nausea and vomiting. **(C)** Drowsiness. **(D)** Hallucination. **(E)** Tremor.

Esketamine significantly increased dizziness incidence [10 studies (11, 15, 17, 18, 20–25); RR: 2.30, 95% CI: 1.75–3.02], particularly with higher doses (≥0.3 mg/kg). No significant associations emerged for nausea/vomiting [9 studies (12, 14, 17–21, 24, 25); RR: 1.12, 95% CI: 0.82–1.53] or drowsiness [4 studies (18, 22, 24, 25); RR: 1.05, 95% CI: 0.70–1.58]. Insufficient evidence existed for hallucinations and tremors (2 studies each), with forest plot details presented in Figure 7.

Heart rate (HR) and mean arterial pressure (MAP) showed clinically relevant elevations during endoscopic instrumentation. Peripheral oxygen saturation (SpO₂) remained within the normal physiological range at all time points, with only minor variations. The complete hemodynamic data can be found in Supplementary material S4.

The meta-analysis showed no significant intergroup differences in procedure time and PACU discharge time. In contrast, orientation recovery time, PADSS scores, patient satisfaction scores, and VAS scores at awakening could not be analyzed due to insufficient data. Details are provided in Supplementary material S5.

### Subgroup analysis

**Figure 8:**
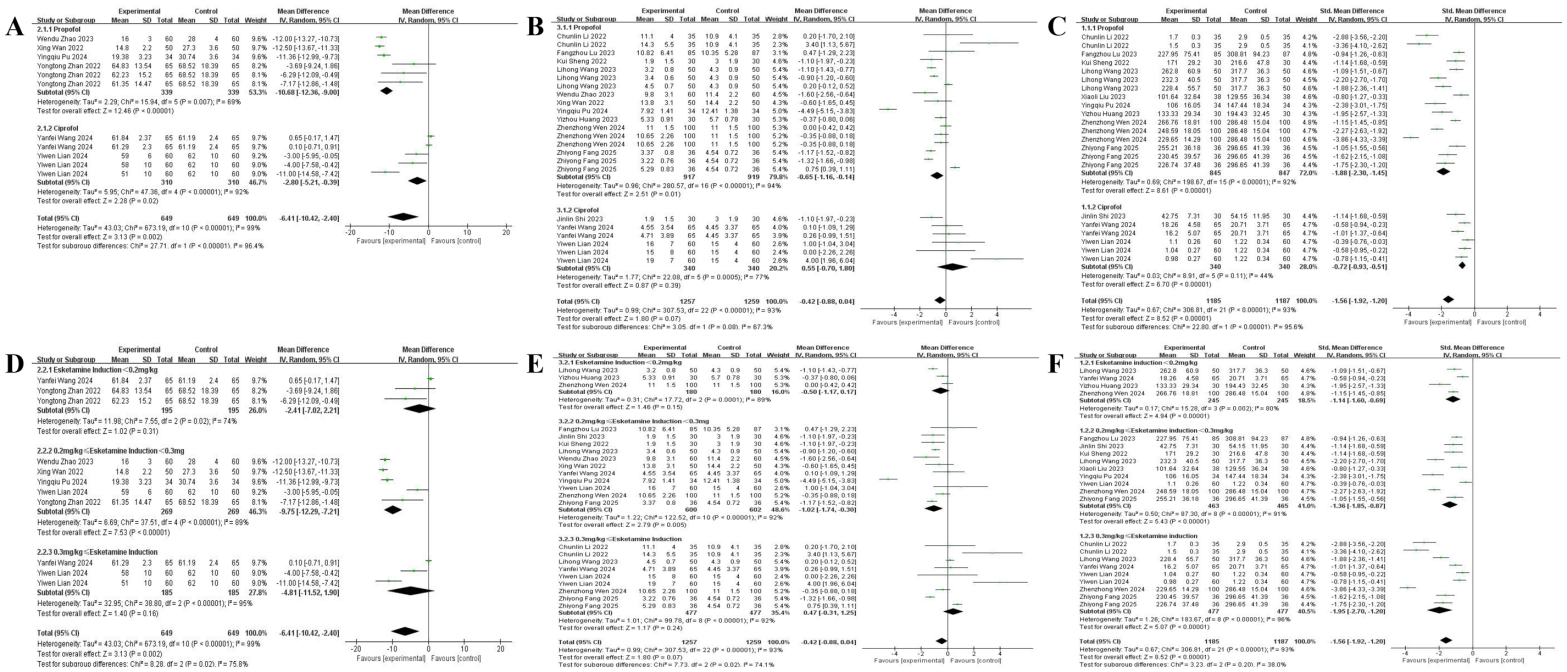
Subgroup analysis of the efficacy endpoints. Effects of esketamine in combination with different sedative agents on. **(A)** Anesthesia onset time. **(B)** Recovery time. **(C)** Sedative requirements; effects of different dosage ranges of esketamine in combination with a sedative on. **(D)** Anesthesia onset time. **(E)** Recovery time. **(F)** Sedative requirements.

Stratified analyses revealed significant differential effects based on sedative selection and esketamine dosing: propofol-based regimens were associated with substantially greater reductions in anesthesia onset time (MD: −10.68 min; 95% CI: −12.36 to −9.00) than ciprofol combinations (MD: −2.80 min; 95% CI: −5.21 to −0.39). Dose-specific examination in onset time was observed only with the 0.2–0.3 mg/kg esketamine dose (MD: −9.75 min; 95% CI: −12.29 to −7.21). Similarly, propofol groups showed a modest reduction in recovery time (MD: −0.65 min; 95% CI: −1.16 to −0.14), an effect not seen with ciprofol. The sedative-sparing effect was also more pronounced with propofol (SMD: −1.88; 95% CI: −2.30 to −1.45) than with ciprofol (SMD: −0.72; 95% CI: −0.93 to −0.51), with all esketamine doses reducing requirements in a clear dose–response manner. For safety, esketamine significantly reduced respiratory depression risk in propofol-based sedation but showed no significant effect in ciprofol groups. Dose-stratified analysis indicated respiratory protection occurred primarily within the 0.2–0.3 mg/kg range. In contrast, the risk of dizziness exhibited a strong dose dependency, increasing significantly at 0.3 mg/kg. Furthermore, esketamine combined with propofol or ciprofol can significantly reduce the incidence of choking cough and body movement, and the greater the dose, the more pronounced the effect. Representative results for selected joints are presented in Figure 8. Detailed findings for all joints are provided in Supplementary material S6.

### Publication bias and sensitivity analysis

Significant publication bias was detected across primary outcomes through funnel plot asymmetry and Egger regression testing, with notable effects for anesthesia onset time (Egger’s *p* = 0.003), recovery time (*p* = 0.008), and sedative requirements (*p* < 0.001). To evaluate result robustness, leave-one-out sensitivity analyses were systematically conducted. For anesthesia onset time, the significant reduction persisted across all iterations (MD: −7.16 to −5.81 min; 95% CI: −11.59 to −1.68), indicating stable treatment effects regardless of individual study removal. Similarly, the substantial sedative-sparing effect remained consistent (SMD: −1.62 to −1.44; 95% CI: −1.98 to −1.13), confirming the reliability of this finding. Three studies employing supratherapeutic esketamine doses (≥0.4 mg/kg)—specifically Chun-lin et al. (12) Group B (0.5 mg/kg), Zhiyong et al. (20) Group C (0.4 mg/kg), and Yiwen et al. (24) Group E3 (0.4 mg/kg)—were identified as disproportionately influencing recovery time estimates. Exclusion of these outliers revealed a statistically significant recovery acceleration effect (MD: −0.74 min; 95% CI: −1.17 to −0.31; *p* = 0.0008), with consistent results across sensitivity iterations (MD: −0.81 to −0.57 min; 95% CI: −1.87 to −0.23). This pattern suggests that esketamine’s recovery benefits become clinically detectable when analysis is restricted to clinically appropriate dosing regimens (≤0.3 mg/kg). Although residual publication bias persists, the directional consistency and magnitude stability across sensitivity analyses strengthen confidence in the overall conclusions. The results are shown in Supplementary material S7.

## Discussion

This systematic review and meta-analysis synthesizes evidence from 15 randomized controlled trials investigating esketamine as an adjunctive agent for sedation during painless gastrointestinal endoscopy. The findings demonstrate that esketamine co-administration significantly reduces total sedative consumption, shortens anesthetic onset time, and mitigates several procedure-related adverse events compared to conventional sedative regimens. Crucially, subgroup analyses identify a therapeutic window of 0.2–0.3 mg/kg esketamine that optimizes clinical benefits while minimizing adverse neuropsychiatric effects.

Esketamine exerts its effects through multiple molecular targets (26). Its primary mechanisms for anesthesia and analgesia involve non-competitive antagonism of NMDA receptors, inhibiting glutamate-mediated neurotransmission in GABAergic pathways. This leads to altered neuronal excitability in cortical and limbic systems, ultimately resulting in loss of consciousness (27). The minimum effective plasma concentration of esketamine for general anesthesia is 0.3 mg/L (28). When used as an adjunct in general anesthesia, a single intravenous dose of 0.2–0.3 mg/kg achieves this target concentration (29). Our study demonstrates that esketamine at 0.2–0.3 mg/kg significantly shortens anesthesia onset time, potentially indicating synergistic interactions at specific NMDA receptor saturation thresholds: 1. At doses <0.2 mg/kg, insufficient NMDA receptor blockade results in inadequate analgesia/sedation, delayed onset, and requires more rescue sedatives. 2. Doses of 0.2–0.3 mg/kg provide adequate NMDA receptor antagonism to achieve the optimal synergy threshold for gastrointestinal endoscopy without causing significant sympathetic activation or psychiatric side effects. 3. At doses ≥0.3 mg/kg, although enhanced receptor blockade occurs, there’s increased incidence of sympathetic overactivation (such as tachycardia and hypertension) and psychiatric side effects (such as dizziness), consistent with our findings.

Ciprofol, similar to propofol, exerts its sedative-hypnotic effects through GABA receptors, yet demonstrates higher liposolubility and potency than propofol (30). Its onset of action occurs within 1–2 min after administration, with gradual recovery within 10 to 18 min, indicating rapid onset characteristics (31, 32). Notably, our study revealed that the propofol-esketamine combination produced a more pronounced acceleration of anesthetic onset. Differences in induction time may be associated with physiological changes—such as decreased body water content and increased fat proportion—which subsequently alter drug distribution volume and modify central nervous system sensitivity. All study groups across the included trials exhibited comparable baseline characteristics (including gender, age, BMI, and ASA classification). We posit that drug dosage constitutes the primary source of heterogeneity. When comparing the sedation efficacy of ciprofol and propofol, a 0.4 mg/kg dose of ciprofol proved to be equal to a 2.0 mg/kg dose of propofol (33). In conclusion, we contend that our findings hold meaningful reference value.

While the primary analysis showed no statistically significant reduction in overall recovery time, sensitivity analyses excluding studies using supratherapeutic esketamine doses (≥0.4 mg/kg) revealed clinically meaningful recovery acceleration in the 0.2–0.3 mg/kg range (34). This underscores the importance of dose optimization (3), as higher doses may prolong recovery through residual NMDA receptor modulation, whereas the recommended ketamine dose of 0.5 mg/kg (3) would equate to approximately 0.25 mg/kg of the more potent esketamine.

The substantial sedative-sparing effect observed—with esketamine reducing cumulative sedative requirements by 25–30%—represents a significant pharmacoeconomic and safety advantage given that conventional intravenous sedatives lack analgesic properties and require higher doses that increase cardiorespiratory risks (35). This effect likely stems from esketamine’s dual sedative-analgesic properties (36), which deepen sedation levels and reduce supplemental dosing needs during painful stimuli like scope insertion, thereby lowering risks associated with high-dose sedative exposure (37).

Regarding safety, esketamine significantly improved intraoperative stability by reducing hypotension and bradycardia through catecholamine reuptake inhibition (38), while decreasing hypoxemia incidence by preserving CO_2_ sensitivity and respiratory drive (39). These mechanisms also reduced coughing and body movements through enhanced analgesia (36). However, sympathomimetic effects increased hypertension and tachycardia incidence (38). Although these conditions are transient, high doses should be avoided in patients with hypertension or severe ischemic heart disease. Postoperatively, esketamine increased dizziness risk—particularly at doses ≥0.3 mg/kg—likely due to its dissociative properties (27), while propofol’s GABAergic activity (40) appeared insufficient to fully counter this effect. No significant associations emerged for nausea/vomiting or somnolence. Furthermore, while increased salivation and laryngospasm are known complications of high-dose esketamine, the trials in this meta-analysis did not demonstrate an increase in the incidence of laryngospasm with esketamine use, a finding that helps alleviate concerns among anesthesiologists.

Several limitations warrant acknowledgment. The geographical concentration of included trials in China may limit generalizability to other populations (41). Significant methodological heterogeneity existed in outcome definitions and sedation protocols (34). Statistical indicators suggested potential publication bias for primary outcomes, though sensitivity analyses supported robustness. Key endpoints like long-term neurocognitive effects remain understudied (42). Most included studies exhibited unclear or high risk of bias in critical domains like allocation concealment.

In conclusion, this meta-analysis supports esketamine (0.2–0.3 mg/kg) as an effective adjunct for painless gastrointestinal endoscopy—the gold-standard diagnostic approach for digestive disorders (41). When combined with propofol or ciprofol, it reduces sedative requirements, accelerates induction, shortens recovery within the therapeutic dose window, and decreases intraoperative cardiorespiratory instability. The trade-offs—transient hemodynamic fluctuations and dose-dependent dizziness—appear manageable at recommended doses. We advocate for 0.2–0.3 mg/kg esketamine as the optimal strategy. Future multinational trials should validate these findings in diverse populations.

## Data Availability

The original contributions presented in the study are included in the article/Supplementary material, further inquiries can be directed to the corresponding author.
