# Supplementary material for: Efficacy and safety of esketamine in patients undergoing painless gastrointestinal endoscopy: a systematic review and meta-analysis of randomized controlled trials
Source: Front Med (Lausanne). 2025 Nov 12;12:1669499. doi: 10.3389/fmed.2025.1669499 (PMC12647098; doi:10.3389/fmed.2025.1669499)
Supplement: Supplementary file 1 [file Table_1.doc]

**Supplementary Appendix 1: Search Strategy**

Part1: PubMed

| #3 | 135 | #1 AND #2   Search: #1 AND #2  ("esketamine"[Supplementary Concept] OR "esketamine"[All Fields] OR "esketamine"[All Fields] OR ("esketamine"[Supplementary Concept] OR "esketamine"[All Fields] OR "l ketamine"[All Fields]) OR ("esketamine"[Supplementary Concept] OR "esketamine"[All Fields] OR "ketamine"[All Fields] OR "ketamine"[MeSH Terms] OR "ketamin"[All Fields] OR "ketamine s"[All Fields] OR "ketamines"[All Fields]) OR ("esketamine"[Supplementary Concept] OR "esketamine"[All Fields] OR "s ketamine"[All Fields]) OR ((((("S"[All Fields] AND "2"[All Fields]) AND "o-chlorophenyl"[All Fields]) AND "2"[All Fields]) AND "methylamino"[All Fields]) AND ("cyclohexanone"[Supplementary Concept] OR "cyclohexanone"[All Fields] OR "cyclohexanones"[MeSH Terms] OR "cyclohexanones"[All Fields])) OR ("esketamine"[Supplementary Concept] OR "esketamine"[All Fields]) OR ("esketamine"[Supplementary Concept] OR "esketamine"[All Fields] OR "esketamine"[All Fields] OR "spravato"[All Fields])) AND ("endoscopy, gastrointestinal"[MeSH Terms] OR ("endoscopy, gastrointestinal"[MeSH Terms] OR ("endoscopy"[All Fields] AND "gastrointestinal"[All Fields]) OR "gastrointestinal endoscopy"[All Fields] OR "endoscopy gastrointestinal"[All Fields]) OR ("endoscopy, gastrointestinal"[MeSH Terms] OR ("endoscopy"[All Fields] AND "gastrointestinal"[All Fields]) OR "gastrointestinal endoscopy"[All Fields] OR ("endoscopies"[All Fields] AND "gastrointestinal"[All Fields])) OR ("endoscopy, gastrointestinal"[MeSH Terms] OR ("endoscopy"[All Fields] AND "gastrointestinal"[All Fields]) OR "gastrointestinal endoscopy"[All Fields] OR ("gastrointestinal"[All Fields] AND "endoscopies"[All Fields]) OR "gastrointestinal endoscopies"[All Fields]) OR ("endoscopy, gastrointestinal"[MeSH Terms] OR ("endoscopy"[All Fields] AND "gastrointestinal"[All Fields]) OR "gastrointestinal endoscopy"[All Fields] OR ("gastrointestinal"[All Fields] AND "endoscopy"[All Fields])) OR ("endoscopy, gastrointestinal"[MeSH Terms] OR ("endoscopy"[All Fields] AND "gastrointestinal"[All Fields]) OR "gastrointestinal endoscopy"[All Fields] OR ("surgical"[All Fields] AND "procedures"[All Fields] AND "endoscopic"[All Fields] AND "gastrointestinal"[All Fields])) OR ("endoscopy, gastrointestinal"[MeSH Terms] OR ("endoscopy"[All Fields] AND "gastrointestinal"[All Fields]) OR "gastrointestinal endoscopy"[All Fields] OR ("endoscopic"[All Fields] AND "gastrointestinal"[All Fields] AND "surgery"[All Fields]) OR "endoscopic gastrointestinal surgery"[All Fields]) OR ("endoscopy, gastrointestinal"[MeSH Terms] OR ("endoscopy"[All Fields] AND "gastrointestinal"[All Fields]) OR "gastrointestinal endoscopy"[All Fields] OR ("endoscopic"[All Fields] AND "gastrointestinal"[All Fields] AND "surgeries"[All Fields])) OR ("endoscopy, gastrointestinal"[MeSH Terms] OR ("endoscopy"[All Fields] AND "gastrointestinal"[All Fields]) OR "gastrointestinal endoscopy"[All Fields] OR ("gastrointestinal"[All Fields] AND "surgeries"[All Fields] AND "endoscopic"[All Fields])) OR ("endoscopy, gastrointestinal"[MeSH Terms] OR ("endoscopy"[All Fields] AND "gastrointestinal"[All Fields]) OR "gastrointestinal endoscopy"[All Fields] OR ("gastrointestinal"[All Fields] AND "surgery"[All Fields] AND "endoscopic"[All Fields]) OR "gastrointestinal surgery endoscopic"[All Fields]) OR ("endoscopy, gastrointestinal"[MeSH Terms] OR ("endoscopy"[All Fields] AND "gastrointestinal"[All Fields]) OR "gastrointestinal endoscopy"[All Fields] OR ("surgeries"[All Fields] AND "endoscopic"[All Fields] AND "gastrointestinal"[All Fields])) OR ("endoscopy, gastrointestinal"[MeSH Terms] OR ("endoscopy"[All Fields] AND "gastrointestinal"[All Fields]) OR "gastrointestinal endoscopy"[All Fields] OR ("surgery"[All Fields] AND "endoscopic"[All Fields] AND "gastrointestinal"[All Fields])) OR ("endoscopy, gastrointestinal"[MeSH Terms] OR ("endoscopy"[All Fields] AND "gastrointestinal"[All Fields]) OR "gastrointestinal endoscopy"[All Fields] OR ("endoscopic"[All Fields] AND "gastrointestinal"[All Fields] AND "surgical"[All Fields] AND "procedures"[All Fields]) OR "endoscopic gastrointestinal surgical procedures"[All Fields]) OR ("endoscopy, gastrointestinal"[MeSH Terms] OR ("endoscopy"[All Fields] AND "gastrointestinal"[All Fields]) OR "gastrointestinal endoscopy"[All Fields] OR ("gastrointestinal"[All Fields] AND "endoscopic"[All Fields] AND "surgical"[All Fields] AND "procedures"[All Fields]) OR "gastrointestinal endoscopic surgical procedures"[All Fields]) OR ("endoscopy, gastrointestinal"[MeSH Terms] OR ("endoscopy"[All Fields] AND "gastrointestinal"[All Fields]) OR "gastrointestinal endoscopy"[All Fields] OR ("procedure"[All Fields] AND "endoscopic"[All Fields] AND "gastrointestinal"[All Fields] AND "surgical"[All Fields])) OR ("endoscopy, gastrointestinal"[MeSH Terms] OR ("endoscopy"[All Fields] AND "gastrointestinal"[All Fields]) OR "gastrointestinal endoscopy"[All Fields] OR ("procedure"[All Fields] AND "gastrointestinal"[All Fields] AND "endoscopic"[All Fields] AND "surgical"[All Fields])) OR ("endoscopy, gastrointestinal"[MeSH Terms] OR ("endoscopy"[All Fields] AND "gastrointestinal"[All Fields]) OR "gastrointestinal endoscopy"[All Fields] OR ("procedures"[All Fields] AND "endoscopic"[All Fields] AND "gastrointestinal"[All Fields] AND "surgical"[All Fields])) OR ("endoscopy, gastrointestinal"[MeSH Terms] OR ("endoscopy"[All Fields] AND "gastrointestinal"[All Fields]) OR "gastrointestinal endoscopy"[All Fields] OR ("procedures"[All Fields] AND "gastrointestinal"[All Fields] AND "endoscopic"[All Fields] AND "surgical"[All Fields])) OR ("endoscopy, gastrointestinal"[MeSH Terms] OR ("endoscopy"[All Fields] AND "gastrointestinal"[All Fields]) OR "gastrointestinal endoscopy"[All Fields] OR ("surgical"[All Fields] AND "procedure"[All Fields] AND "endoscopic"[All Fields] AND "gastrointestinal"[All Fields]))) |
| --- | --- | --- |
| #2 | 148,572 | #2"endoscopy, gastrointestinal"[MeSH Terms] OR ("endoscopy, gastrointestinal"[MeSH Terms] OR ("endoscopy"[All Fields] AND "gastrointestinal"[All Fields]) OR "gastrointestinal endoscopy"[All Fields] OR "endoscopy gastrointestinal"[All Fields]) OR ("endoscopy, gastrointestinal"[MeSH Terms] OR ("endoscopy"[All Fields] AND "gastrointestinal"[All Fields]) OR "gastrointestinal endoscopy"[All Fields] OR ("endoscopies"[All Fields] AND "gastrointestinal"[All Fields])) OR ("endoscopy, gastrointestinal"[MeSH Terms] OR ("endoscopy"[All Fields] AND "gastrointestinal"[All Fields]) OR "gastrointestinal endoscopy"[All Fields] OR ("gastrointestinal"[All Fields] AND "endoscopies"[All Fields]) OR "gastrointestinal endoscopies"[All Fields]) OR ("endoscopy, gastrointestinal"[MeSH Terms] OR ("endoscopy"[All Fields] AND "gastrointestinal"[All Fields]) OR "gastrointestinal endoscopy"[All Fields] OR ("gastrointestinal"[All Fields] AND "endoscopy"[All Fields])) OR ("endoscopy, gastrointestinal"[MeSH Terms] OR ("endoscopy"[All Fields] AND "gastrointestinal"[All Fields]) OR "gastrointestinal endoscopy"[All Fields] OR ("surgical"[All Fields] AND "procedures"[All Fields] AND "endoscopic"[All Fields] AND "gastrointestinal"[All Fields])) OR ("endoscopy, gastrointestinal"[MeSH Terms] OR ("endoscopy"[All Fields] AND "gastrointestinal"[All Fields]) OR "gastrointestinal endoscopy"[All Fields] OR ("endoscopic"[All Fields] AND "gastrointestinal"[All Fields] AND "surgery"[All Fields]) OR "endoscopic gastrointestinal surgery"[All Fields]) OR ("endoscopy, gastrointestinal"[MeSH Terms] OR ("endoscopy"[All Fields] AND "gastrointestinal"[All Fields]) OR "gastrointestinal endoscopy"[All Fields] OR ("endoscopic"[All Fields] AND "gastrointestinal"[All Fields] AND "surgeries"[All Fields])) OR ("endoscopy, gastrointestinal"[MeSH Terms] OR ("endoscopy"[All Fields] AND "gastrointestinal"[All Fields]) OR "gastrointestinal endoscopy"[All Fields] OR ("gastrointestinal"[All Fields] AND "surgeries"[All Fields] AND "endoscopic"[All Fields])) OR ("endoscopy, gastrointestinal"[MeSH Terms] OR ("endoscopy"[All Fields] AND "gastrointestinal"[All Fields]) OR "gastrointestinal endoscopy"[All Fields] OR ("gastrointestinal"[All Fields] AND "surgery"[All Fields] AND "endoscopic"[All Fields]) OR "gastrointestinal surgery endoscopic"[All Fields]) OR ("endoscopy, gastrointestinal"[MeSH Terms] OR ("endoscopy"[All Fields] AND "gastrointestinal"[All Fields]) OR "gastrointestinal endoscopy"[All Fields] OR ("surgeries"[All Fields] AND "endoscopic"[All Fields] AND "gastrointestinal"[All Fields])) OR ("endoscopy, gastrointestinal"[MeSH Terms] OR ("endoscopy"[All Fields] AND "gastrointestinal"[All Fields]) OR "gastrointestinal endoscopy"[All Fields] OR ("surgery"[All Fields] AND "endoscopic"[All Fields] AND "gastrointestinal"[All Fields])) OR ("endoscopy, gastrointestinal"[MeSH Terms] OR ("endoscopy"[All Fields] AND "gastrointestinal"[All Fields]) OR "gastrointestinal endoscopy"[All Fields] OR ("endoscopic"[All Fields] AND "gastrointestinal"[All Fields] AND "surgical"[All Fields] AND "procedures"[All Fields]) OR "endoscopic gastrointestinal surgical procedures"[All Fields]) OR ("endoscopy, gastrointestinal"[MeSH Terms] OR ("endoscopy"[All Fields] AND "gastrointestinal"[All Fields]) OR "gastrointestinal endoscopy"[All Fields] OR ("gastrointestinal"[All Fields] AND "endoscopic"[All Fields] AND "surgical"[All Fields] AND "procedures"[All Fields]) OR "gastrointestinal endoscopic surgical procedures"[All Fields]) OR ("endoscopy, gastrointestinal"[MeSH Terms] OR ("endoscopy"[All Fields] AND "gastrointestinal"[All Fields]) OR "gastrointestinal endoscopy"[All Fields] OR ("procedure"[All Fields] AND "endoscopic"[All Fields] AND "gastrointestinal"[All Fields] AND "surgical"[All Fields])) OR ("endoscopy, gastrointestinal"[MeSH Terms] OR ("endoscopy"[All Fields] AND "gastrointestinal"[All Fields]) OR "gastrointestinal endoscopy"[All Fields] OR ("procedure"[All Fields] AND "gastrointestinal"[All Fields] AND "endoscopic"[All Fields] AND "surgical"[All Fields])) OR ("endoscopy, gastrointestinal"[MeSH Terms] OR ("endoscopy"[All Fields] AND "gastrointestinal"[All Fields]) OR "gastrointestinal endoscopy"[All Fields] OR ("procedures"[All Fields] AND "endoscopic"[All Fields] AND "gastrointestinal"[All Fields] AND "surgical"[All Fields])) OR ("endoscopy, gastrointestinal"[MeSH Terms] OR ("endoscopy"[All Fields] AND "gastrointestinal"[All Fields]) OR "gastrointestinal endoscopy"[All Fields] OR ("procedures"[All Fields] AND "gastrointestinal"[All Fields] AND "endoscopic"[All Fields] AND "surgical"[All Fields])) OR ("endoscopy, gastrointestinal"[MeSH Terms] OR ("endoscopy"[All Fields] AND "gastrointestinal"[All Fields]) OR "gastrointestinal endoscopy"[All Fields] OR ("surgical"[All Fields] AND "procedure"[All Fields] AND "endoscopic"[All Fields] AND "gastrointestinal"[All Fields])) |
| #1 | [27,33](https://pubmed.ncbi.nlm.nih.gov/?term=((((((((Esketamine[MeSH+Terms]))+OR+(Esketamine))+OR+(L-Ketamine))+OR+((-)-Ketamine))+OR+(S-Ketamine))+OR+((S)-2-(o-chlorophenyl)-2-(methylamino)cyclohexanone))+OR+(Kataved))+OR+(Spravato)&sort=)0 | #1"esketamine"[Supplementary Concept] OR "esketamine"[All Fields] OR "esketamine"[All Fields] OR ("esketamine"[Supplementary Concept] OR "esketamine"[All Fields] OR "l ketamine"[All Fields]) OR ("esketamine"[Supplementary Concept] OR "esketamine"[All Fields] OR "ketamine"[All Fields] OR "ketamine"[MeSH Terms] OR "ketamin"[All Fields] OR "ketamine s"[All Fields] OR "ketamines"[All Fields]) OR ("esketamine"[Supplementary Concept] OR "esketamine"[All Fields] OR "s ketamine"[All Fields]) OR ((((("S"[All Fields] AND "2"[All Fields]) AND "o-chlorophenyl"[All Fields]) AND "2"[All Fields]) AND "methylamino"[All Fields]) AND ("cyclohexanone"[Supplementary Concept] OR "cyclohexanone"[All Fields] OR "cyclohexanones"[MeSH Terms] OR "cyclohexanones"[All Fields])) OR ("esketamine"[Supplementary Concept] OR "esketamine"[All Fields]) OR ("esketamine"[Supplementary Concept] OR "esketamine"[All Fields] OR "esketamine"[All Fields] OR "spravato"[All Fields]) |

Part2: Web of science

| #3 | 98 | #2 AND #1 |
| --- | --- | --- |
| #2 | 11,721 | TS=("Endoscopy, Gastrointestinal" OR "Endoscopies, Gastrointestinal" OR "Gastrointestinal Endoscopies" OR "Gastrointestinal Endoscopy" OR "Surgical Procedures, Endoscopic Gastrointestinal" OR "Endoscopic Gastrointestinal Surgery" OR "Endoscopic Gastrointestinal Surgeries"OR "Gastrointestinal Surgeries, Endoscopic"OR "Gastrointestinal Surgery, Endoscopic"OR "Surgeries, Endoscopic Gastrointestinal"OR "Surgery, Endoscopic Gastrointestinal"OR "Endoscopic Gastrointestinal Surgical Procedures"OR "Gastrointestinal Endoscopic Surgical Procedures"OR "Procedure, Endoscopic Gastrointestinal, Surgical"OR "Procedure, Gastrointestinal Endoscopic Surgical"OR "Procedures, Endoscopic Gastrointestinal, Surgical"OR "Procedures, Gastrointestinal Endoscopic Surgical"OR "Surgical Procedure, Endoscopic Gastrointestinal") |
| #1 | 32,161 | TS=("Esketamine" OR "L-Ketamine" OR "(-)-Ketamine" OR "S-Ketamine" OR "(S)-2-(o-chlorophenyl)-2-(methylamino)cyclohexanone" OR "Kataved" OR "Spravato") |

Part3: Embase

| #7 | 33 | #3 AND #6 |
| --- | --- | --- |
| #6 | 224,340 | #4 OR #5 |
| #5 | 15,815 | ('endoscopy, gastrointestinal':ab,ti OR 'gastrointestinal endoscopy':ab,ti) AND [embase]/lim |
| #4 | 224,329 | ('gastrointestinal endoscopy'/exp OR 'gastrointestinal endoscopy') AND [embase]/lim |
| #3 | 2,407 | #1 OR #2 |
| #2 | 2,407 | (esketamine:ab,ti OR 'am 101':ab,ti OR 'am101':ab,ti OR 'cle 100':ab,ti OR 'cle100':ab,ti OR 'cz 06':ab,ti OR 'cz06':ab,ti OR 'esgamda':ab,ti OR 'eskelan':ab,ti OR 'esketamin':ab,ti OR 'esketamine hydrochloride':ab,ti OR 'esketiv':ab,ti OR 'falkieri':ab,ti OR 'jnj 54135419':ab,ti OR 'jnj 5419':ab,ti OR 'jnj54135419':ab,ti OR 'jnj5419':ab,ti OR 'ketanest s':ab,ti OR 'keyzilen':ab,ti OR 'pg 061':ab,ti OR 'pg061':ab,ti OR 's ketamin':ab,ti OR 's ketamine':ab,ti OR 's-ketamin':ab,ti OR 'sinmelan':ab,ti OR 'spravato':ab,ti OR 'vesierra':ab,ti OR 'esketamine':ab,ti) AND [embase]/lim |
| #1 | 1,409 | esketamine:ab,ti AND [embase]/lim |

Part4: Cochrane

| #10 | 46 | #3 and #9 |
| --- | --- | --- |
| #9 | 10548 | #4 OR #5 OR #6 OR #7 OR #8 |
| #8 | 1315 | (Procedures, Endoscopic Gastrointestinal, Surgical):ti,ab,kw OR (Endoscopic Gastrointestinal Surgical Procedures):ti,ab,kw OR (Gastrointestinal Surgery, Endoscopic):ti,ab,kw |
| #7 | 1364 | (Surgery, Endoscopic Gastrointestinal):ti,ab,kw OR (Surgeries, Endoscopic Gastrointestinal):ti,ab,kw OR (Surgical Procedures, Endoscopic Gastrointestinal):ti,ab,kw OR (Gastrointestinal Endoscopic Surgical Procedures):ti,ab,kw OR (Procedure, Gastrointestinal Endoscopic Surgical):ti,ab,kw |
| #6 | 311 | (Procedure, Endoscopic Gastrointestinal, Surgical):ti,ab,kw OR (Gastrointestinal Surgeries, Endoscopic):ti,ab,kw OR (Endoscopic Gastrointestinal Surgeries):ti,ab,kw OR (Procedures, Gastrointestinal Endoscopic Surgical):ti,ab,kw OR (Surgical Procedure, Endoscopic Gastrointestinal):ti,ab,kw |
| #5 | 5759 | (endoscopy, gastrointestinal):ti,ab,kw OR (Synonyms: Gastrointestinal Endoscopy):ti,ab,kw OR (Gastrointestinal Endoscopies):ti,ab,kw OR (Endoscopies, Gastrointestinal):ti,ab,kw OR (Endoscopic Gastrointestinal Surgery):ti,ab,kw |
| #4 | 6384 | MeSH descriptor: [Endoscopy, Gastrointestinal] explode all trees |
| #3 | 1983 | #1 OR #2 |
| #2 | 1973 | (Esketamine):ti,ab,kw OR (Kataved):ti,ab,kw OR (Spravato):ti,ab,kw OR (S-ketamine):ti,ab,kw OR (L-Ketamine):ti,ab,kw |
| #1 | 1525 | Esketamine |

Part5: Chinese National Knowledge Infrastructure

SU%=('艾司氯胺酮'+'盐酸艾司氯胺酮'+'右旋氯胺酮') and SU%=('胃肠镜检查'+'消化内镜检查'+'胃镜检查'+'结肠镜检查'+'无痛内镜')

Part6: Wangfang Database

(主题:(“艾司氯胺酮“ or “盐酸艾司氯胺酮“ or “右旋氯胺酮”) )and (主题:(“胃肠镜检查” or “消化内镜检查” or “胃镜检查” or “结肠镜检查” or “无痛内镜”) )

Part7: Chinese Science and Technology Journal Database

(U=艾司氯胺酮 OR 盐酸艾司氯胺酮 OR 右旋氯胺酮) AND (U=胃肠镜检查 OR 消化内镜检查 OR 胃镜检查 OR 结肠镜检查 OR 无痛内镜)

Part8: Chinese Biomedical Literature Service System

( "艾司氯胺酮"[全部字段] OR "盐酸艾司氯胺酮"[全部字段] OR "右旋氯胺酮"[全部字段]) AND( "胃肠镜检查"[全部字段] OR "消化内镜检查"[全部字段] OR "胃镜检查"[全部字段] OR "结肠镜检查"[全部字段] OR "无痛内镜"[全部字段])

**Supplementary Appendix 2: Study characteristics**

| Study | Methodological characteristics | Gender  (male/female) | Age  (years) | BMI  (kg/m^2^) | ASA | Procedure Type | Intervention | | Outcome measures | | | | | | | | | | |  |
| --- | --- | --- | --- | --- | --- | --- | --- | --- | --- | --- | --- | --- | --- | --- | --- | --- | --- | --- | --- | --- |
|  |  |  |  |  |  |  | Control Group Regimen | Supplemental Sedative Dosing | HR | SBP | D  B  P | MAP | S  PO_2_ | R  R | B  I  S | Time | Sedative Dosing Details | AssessmentScales | Procedure-Related Adverse Events | Postoperative Complications |
| Xing Wan,2022 | RCT | C: 24/26, E:  29/21 | C:  51.5±14.4  E:  52.4±11.6 | C:  24.3±3.6,  E:  23.9±2.3 | Ⅰ-Ⅱ | GE | C: iv, PPF 2.0-2.5 mg/kg + ivp, PPF 4-6mg/kg·h  E: iv, esketamine 0.25 mg/kg + iv, PPF 2.0-2.5 mg/kg + ivp, PPF 4-6 mg/kg·h | iv, PPF 0.5-1.0 mg/kg | T1  T2  T6 | T1  T2  T6 | T1  T2  T6 |  | T1  T2  T6 |  |  | AB  DE | CD | AB | EFGH | A |
| Chunlin Li,2022 | RCT | C: 22/13, E1: 21/14, E2: 22/13 | C: 46.1±12.1, E1: 45.8±11.6, E2: 43.5±11.5 | C:  21.6±2.3, E1: 21.2±2.6, E2:  22.6±2.7 | Ⅰ-Ⅱ | G | C: iv, NS+iv, PPF 20-40mg/10s,  E1: iv, esketamine 0.3mg/kg+iv,PPF 20-40mg/10s,  E2: iv, esketamine 0.5mg/kg+iv,PPF 20-40mg/10s | iv,PPF 20-50 mg | T1  T6 |  |  | T1  T6 | T1  T6 |  |  | BE | E | B | ABCDH | BE |
| Kui Sheng,2022 | RCT | C: 20/10, E:  15/15 | C: 43.7±13.3,  E:  45.2±9.2 | C:  22.3±3.8,  E:  23.9±2.4 | Ⅰ-Ⅱ | GE | C:iv,NS 10ml+iv,PPF 1-2mg/kg E:iv,esketamine 0.2mg/kg+iv,PPF 1-2mg/kg | iv, PPF 10-20 mg | T0  T2  T3  T4  T5 |  |  | T0  T2  T3  T4  T5 |  |  |  | BDE | E | C | ABEG |  |
| Wendu Zhao,2023 | RCT | C: 43/17, E:  40/20 | C:  70.0±6.0,  E:  68.0±6.0 | C:  22.6±1.0,  E:  22.4±0.6 | Ⅰ-Ⅱ | GE | C:iv,PPF 2.0-2.5mg/kg E:iv,esketamine 0.25mg/kg+iv,PPF 2.0-2.5mg/kg | iv, PPF 0.5-1.0 mg/kg | T0  T2  T6 |  |  | T0  T2  T6 | T0  T2  T6 |  | T0  T2  T6 | AB  DE | CD | A | ABEH | B |
| Fangzhou Lu,2023 | RCT | C: 48/39, E:  51/34 | C: 51.0±10.9,  E:  51.4±10.2 | C:  23.4±2.8,  E:  23.8±2.6 | Ⅰ-Ⅱ | GE | C:iv,NS+iv,PPF 1.5-2.5mg/kg E:iv,esketamine 0.2mg/kg+iv,PPF 1.5-2.5mg/kg | iv, PPF 0.5 mg/kg | T0  T3  T5 |  |  | T0  T3  T5 | T0  T3  T5 |  |  | BE | ADE | H | ABEFG | AB |
| Yizhou Huang,2023 | RCT | C: 19/11, E:  18/12 | C: 50.67±7.21, E: 49.93±5.11 | C: 25.25±3.73,  E: 24.88±3.22 | Ⅰ-Ⅱ | C | C:iv,NS+TCI,PPF 3.0μg/ml E:iv,esketamine 0.15mg/kg+TCI,PPF 3.0μg/ml | TCI, PPF increase 0.5 μg/ml |  |  |  |  |  |  |  | BDE | E | CD | ABE | B |
| Lihong Wang,2023 | RCT | C: 23/27, E1: 24/26, E2: 25/25, E3: 24/26 | C: 49.1±10.7, E1: 51.2±13.5, E2: 49.4±14.4, E3: 50.8±11.6 | C: 22.9±3.7, E1: 23.5±2.3, E2: 24.0±4.1, E3:  23.8±3.6 | Ⅰ-Ⅱ | GE | C:iv,NS+iv,PPF 1-3.5mg/kg  +ivp,PPF 3-5.5mg/kg·h E1:iv,esketamine 0.15mg/kg+iv,PPF 1-3.5mg/kg  +ivp,PPF 3-5.5mg/kg·h E2:iv,esketamine 0.25mg/kg+iv,PPF 1-3.5mg/kg  +ivp,PPF 3-5.5mg/kg·h E3:iv,esketamine 0.35mg/kg+iv,PPF 1-3.5mg/kg  +ivp,PPF 3-5.5mg/kg·h | iv,PPF  0.5-1.0mg/kg | T1  T3  T4  T6 |  |  | T1  T3  T4  T6 | T1  T3  T4  T6 | T1  T3  T4  T6 | T1  T3  T4  T6 | BDE | E | C | ABDEFGH | ABD |
| Zhenzhong Wen,2024 | RCT | C: 47/53, E1: 49/51, E2: 47/53, E3: 48/52 | C: 55.28±19.18, E1: 56.29±18.05, E2: 54.12±19.18, E3: 54.82±17.67 | C: 23.37±2.40,  E1: 23.48±2.55,  E2: 23.73±2.63,  E3: 23.5±2.85 | Ⅰ-Ⅱ | GE | C: iv, NS + TCI, PPF 4.0μg/ml + TCI, PPF 2.5-3.5 μg/ml,  E1: iv, esketamine 0. 1mg/kg + TCI, PPF 4.0μg/ml + TCI, PPF 2.5-3.5 μg/ml,  E2: iv, esketamine 0.2 mg/kg + TCI, PPF 4.0 μg/ml + TCI, PPF 2.5-3.5 μg/ml,  E3: iv, esketamine 0.3 mg/kg + TCI, PPF 4.0 μg/ml + TCI, PPF 2.5-3.5 μg/ml | TCI,PPF increase 0.5 μg/ml | T1  T3  T5  T6 |  |  | T1  T3  T5  T6 | T1  T3  T5  T6 |  |  | BDE | BE | EF | EFGH | ABC |
| Yingqiu Pu 2024 | RCT | C: 18/16, E:  17/17 | C: 49.83±6.61, E: 50.42±5.97 | C: 22.13±4.38,  E: 21.85±5.67 | Ⅰ-Ⅱ | C | C: ivp, PPF 2-3 mg/kg,  E: ivp, esketamine 0.25 mg/kg + ivp, PPF 2-3 mg/kg | iv,PPF 0.5-1.0 mg/kg | T1  T5 |  |  | T1  T5 | T1  T5 |  |  | AB | E | K | BEGH | B |
| Zhiyong Fang,2025 | RCT | C: 20/16, E1: 20/16, E2: 19/17, E3: 22/14 | C: 52.85±8.05, E1: 51.45±8.42, E2: 52.63±8.19, E3: 51.72±7.78 | C: 23.25±2.17,  E1: 23.67±2.14,  E2: 24.12±2.03,  E3: 23.86±2.26 | Ⅰ-Ⅱ | GE | C: iv, NS + iv, PPF 1.5-2.5 mg/kg + ivp, PPF 4-6 mg/kg·h, E1: iv, esketamine 0.2 mg/kg + iv, PPF 1.5-2.5 mg/kg + ivp, PPF 4-6 mg/kg·h, E2: iv, esketamine 0.3 mg/kg + iv, PPF 1.5-2.5 mg/kg + ivp, PPF 4-6 mg/kg·h, E3: iv, esketamine 0.4mg/kg + iv, PPF 1.5-2.5 mg/kg + ivp, PPF 4-6mg/kg·h | iv, PPF 0.5-1.0 mg/kg | T0  T2  T3  T4  T5 |  |  | T0  T2  T3  T4  T5 | T0  T2  T3  T4  T5 |  |  | BD | AE | G | ABDG | AB |
| Yongtong Zhan,2022 | RCT | C: 38/27， E1: 38/27,  E2: 32/33,  E3: 30/35 | C: 44.94±10.031,  E1: 42.71±10.148,  E2: 45.89±9.292, E3: 44.38±10.23 | C: 22.67±2.755  E1: 22.74±2.664  E2: 23.06±2.770  E3: 21.99±2.730 | Ⅰ-Ⅱ | GE | C:iv,NS 0.1ml/kg+iv,PPF 1.5mg/kg E1:iv,esketamine 0.05mg/kg+iv,PPF 1.5mg/kg E2:iv,esketamine 0.1mg/kg+iv,PPF 1.5mg/kg E3:iv,esketamine 0.2mg/kg+iv,PPF 1.5mg/kg | iv, PPF 0.5 mg/kg | T0  T1  T3  T4  T5 | T0  T1  T3  T4  T5 | T0  T1  T3  T4  T5 |  | T0  T1  T3  T4  T5 |  |  | ACE | F | IJ | ACEFGH | ABDE |
| Xiaoli Liu,2023 | RCT | C: 12/26,  E: 16/22, | C: 49.03±10.81, E: 45.68±13.83 | C: 22.40±3.07, E: 21.57±3.02 | Ⅰ-Ⅱ | G | C: iv, NS + iv, PPF 1mg/kg,  E: iv, esketamine 0.2 mg/kg + iv, PPF 1mg/kg | iv, PPF 0.5 mg/kg | T1  T6 |  |  | T1  T6 | T1  T6 |  |  | CE | ADE |  | ABCDGH | ABC |
| Jinlin Shi,2023 | RCT | C: 20/10,  E: 15/15, | C:  68.7±6.7,  E:  69.2±5.6 | C:  25.4±3.8,  E:  26.2±3.0 | Ⅰ-Ⅱ | GE | C: iv, Ciprofol 0.3 mg/kg,  E: iv, Ciprofol 0.3 mg/kg + iv, esketamine 0.2 mg/kg | iv, Ciprofol ≤ 0.15 mg/kg | T0  T2  T3  T4  T5 |  |  | T0  T2  T3  T4  T5 |  |  |  | BCE | E | C | AEGH | A |
| Yiwen Lian,2024 | RCT | C: 22/38, E1: 23/37, E2: 21/39, E3: 22/38 | C: 44.0±11.0, E1: 44.0±11.0, E2: 42.0±10.0, E3: 45.0±11.0 | C:  23.0±4.0, E1: 23.0±3.0, E2: 23.0±4.0, E3:  24.0±3.0 | Ⅰ-Ⅱ | GE | C: iv,Ciprofol 0.4 mg/kg + ivp, Ciprofol 0.8-1.5 mg/kg·h,  E1: iv, esketamine 0.2 mg/kg + iv, Ciprofol 0.2-0.4 mg/kg + ivp, Ciprofol 0.8-1.5 mg/kg·h,  E2: iv, esketamine 0.3 mg/kg + iv, Ciprofol 0.2-0.4mg/kg+ivp,Ciprofol 0.8-1.5mg/kg·h E3:iv,esketamine 0.4mg/kg+iv,Ciprofol 0.2-0.4mg/kg+ivp, Ciprofol 0.8-1.5mg/kg·h | iv, Ciprofol 0.1 mg/kg | T0  T3  T5  T6 |  |  | T0  T3  T5  T6 | T0  T3  T5  T6 | T0  T3  T5  T6 | T0  T3  T5  T6 | AB  DE | EC |  | ABEFG |  |
| Yanfei Wang,2024 | RCT | C: 27/38, E1: 38/27, E2: 35/30 | C: 66.06±5.60, E1: 66.16±5.13, E2: 67.06±4.65 | C: 24.53±2.59,  E1: 24.16±2.60,  E2: 23.70±2.32 | Ⅰ-Ⅱ | GE | C: iv, NS 0.1 ml/kg + iv, Ciprofol 0.3 mg/kg,  E1: iv, esketamine 0.15 mg/kg + iv, Ciprofol 0.3 mg/kg, E2: iv, esketamine 0.30 mg/kg + iv, Ciprofol 0.3 mg/kg | iv,Ciprofol 0.15-0.20 mg/kg | T1  T3  T4  T6 | T1  T3  T4  T6 | T1  T3  T4  T6 |  | T1  T3  T4  T6 |  |  | ABE | ADE |  | AFG | ABD |
| HR SBP  DBP MAP  SPO_2_  RR  BIS | T0: Baseline; T1: Before induction; T2: Pre-gastroscopy insertion; T3: Post-gastroscopy insertion; T4: Pre-colonoscopy insertion; T5: Post-colonoscopy insertion; T6: Awakening | | | | | | | | | | | | | | | | | | | |
| Time | A.Anesthesia Onset Time;B.Recovery Time; C.Orientation recovery time; D.PACU stay time/recovery time; E.Procedure time | | | | | | | | | | | | | | | | | | | |
| Sedative Dosing Details | A.Induction dose; B.Induction target concentration; C.Number of additional attempts; D.Additional dosage; E.Total sedative requirements; F.Per minute consumption | | | | | | | | | | | | | | | | | | | |
| Assessment Scales | A.PADSS; B.VAS(awakening); C.Patient satisfaction; D.Physician operation satisfaction; E.Ramsay; F.STAI; G.QoR-40; H.LiKert; I.MMSE; J.Awakening Status; K.Sedation and analgesia effect | | | | | | | | | | | | | | | | | | | |
| Procedure-Related Adverse Events | A.Hypotension; B.Bradycardia; C.Hypertension; D.Tachycardia; E.Apnea; F.Cough; G.Body movement; H.Injection pain | | | | | | | | | | | | | | | | | | | |
| Postoperative Complications | A.Dizziness; B.Nausea and vomiting; C.Drowsiness; D.Hallucination; E.Tremor | | | | | | | | | | | | | | | | | | |  |

BMI,Body Mass Index; ASA,American Society of Anesthesiologists;

iv,intravenous injection; ivp,intravenous pump; TCI,Target-controlledinfusion;

GE, Gastrointestinal endoscopy; G, Gastroscopy; C, Colonoscopy;

HR,[Heart Rate](https://zhidao.baidu.com/search?word=Heart Rate&fr=iknow_pc_qb_highlight); SBP,Systolic Blood Pressure; DBP,Diastolic Blood Pressure; MAP,Mean Arterial Pressure; SPO_2_,Peripheral capillary oxygen saturation; RR,Respiratory Rate; BIS,Bispectral Index;

PADSS,Post Anaesthetic Discharge Scoring System; VAS,Visual analogue scale; STAI,State-trait anxiety inventory; QoR-40,Quality of Recovery-40 questionnaire; LiKert,Likert scale; MMSE,Mini-Mental State Examination;

**Supplementary Appendix 3: Medication Dosage**


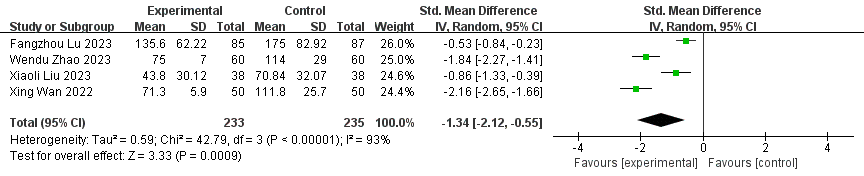


Figure S1: Comparison of supplemental doses between groups.


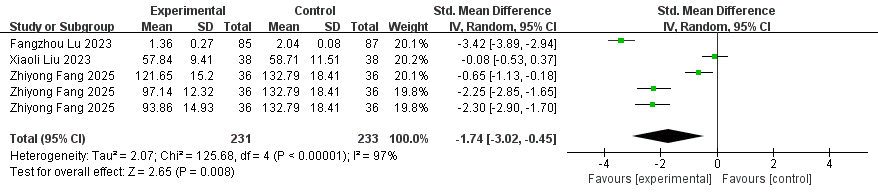


Figure S2: Comparison of induction doses between groups.


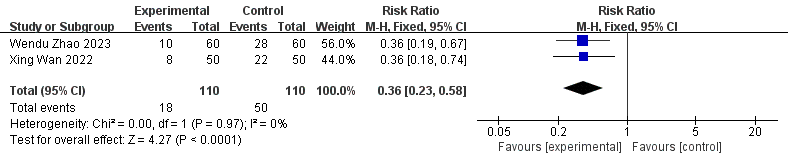


Figure S3: Comparison of the number of supplemental doses between groups.

**Supplementary Appendix 4: Hemodynamic Data**


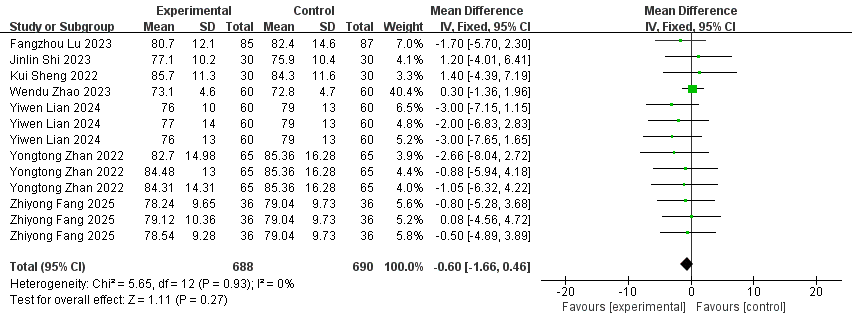
Figure S4: Comparison of heart rate at time T0 (Baseline) between groups.


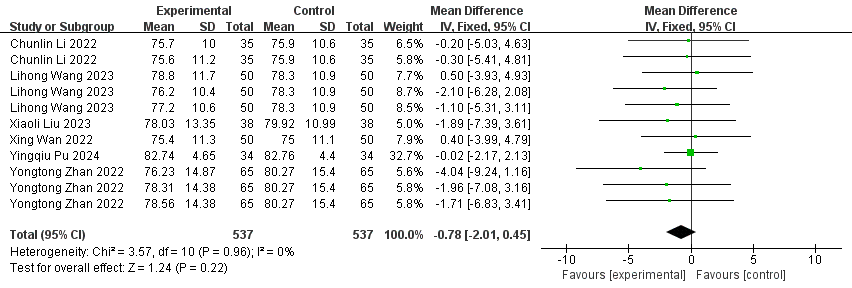
Figure S5: Comparison of heart rate at time T1 (Before induction) between groups.


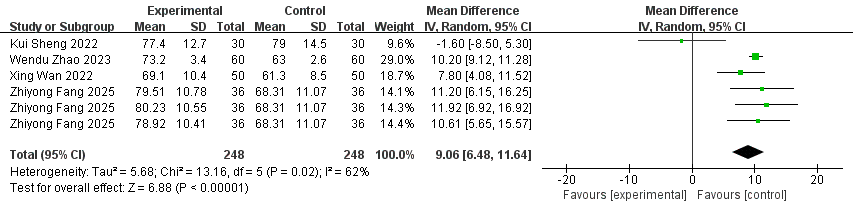
Figure S6: Comparison of heart rate at time T2 (Pre-gastroscopy insertion) between groups.


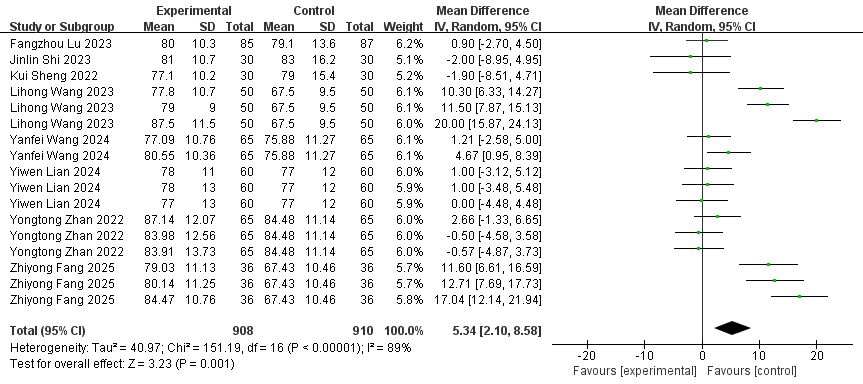


Figure S7: Comparison of heart rate at time T3 (Post-gastroscopy insertion) between groups.


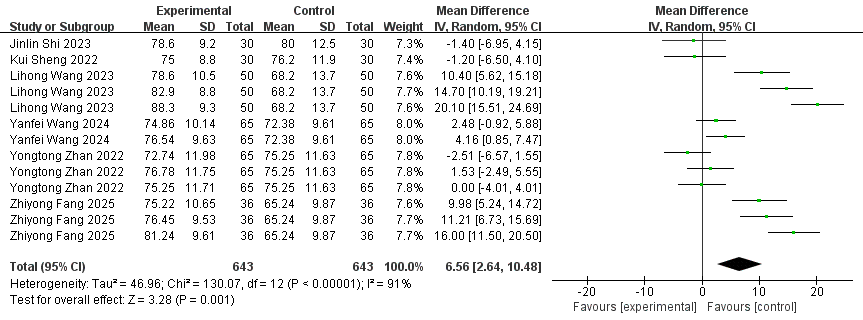


Figure S8: Comparison of heart rate at time T4 (Pre-colonoscopy insertion) between groups.


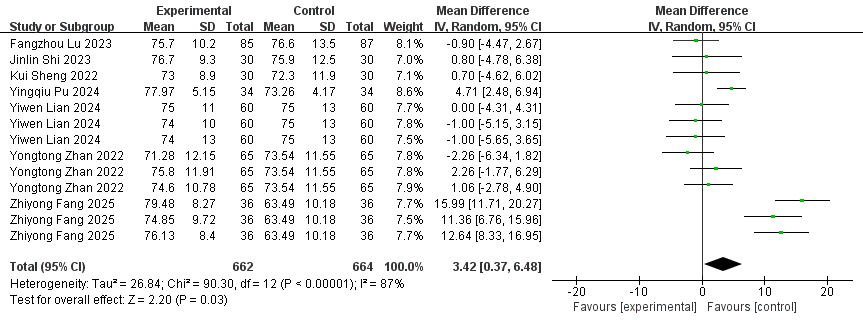


Figure S9: Comparison of heart rate at time T5 (Post-colonoscopy insertion) between groups.


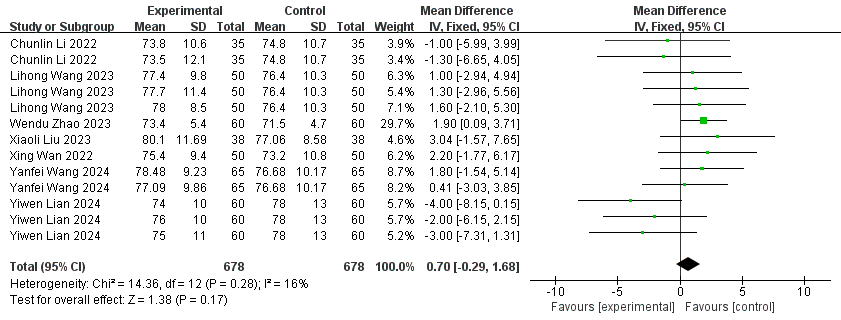


Figure S10: Comparison of heart rate at time T6 (Awakening) between groups.


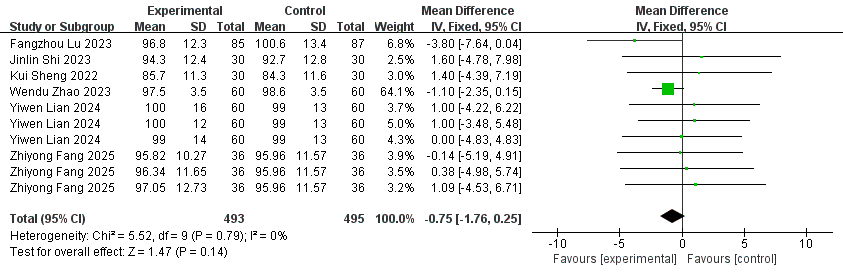


Figure S11: Comparison of mean arterial pressure at time T0 (Baseline) between groups.


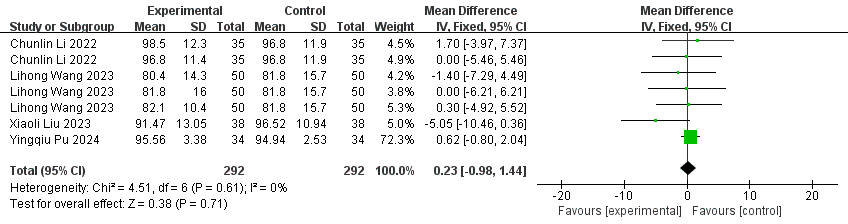


Figure S12: Comparison of mean arterial pressure at time T1(Before induction) between groups.


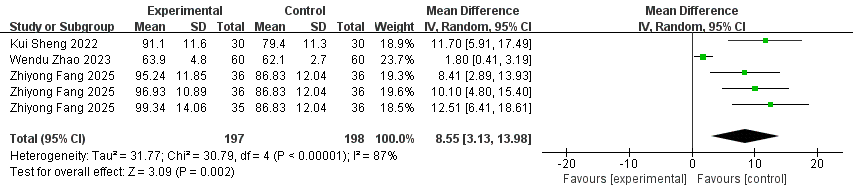


Figure S13: Comparison of mean arterial pressure at time T2 (Pre-gastroscopy insertion) between groups.


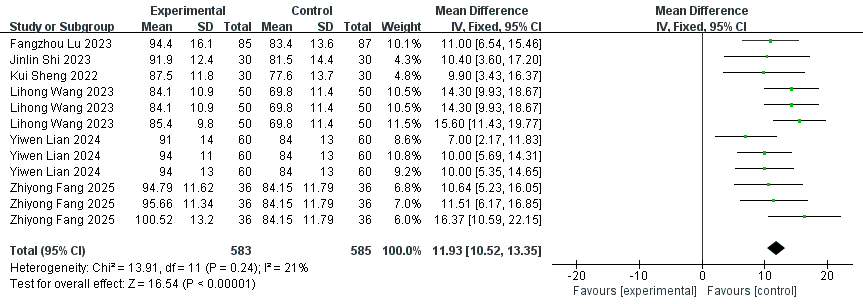


Figure S14: Comparison of mean arterial pressure at time T3 (Post-gastroscopy insertion) between groups.


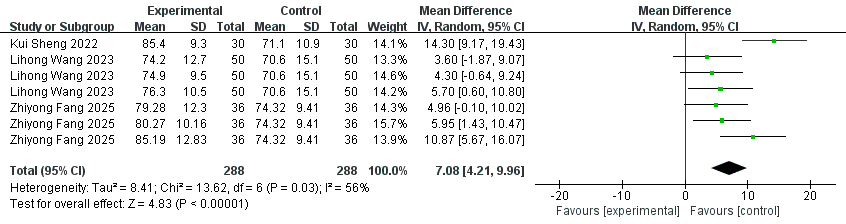


Figure S15: Comparison of mean arterial pressure at time T4 (Pre-colonoscopy insertion) between groups.


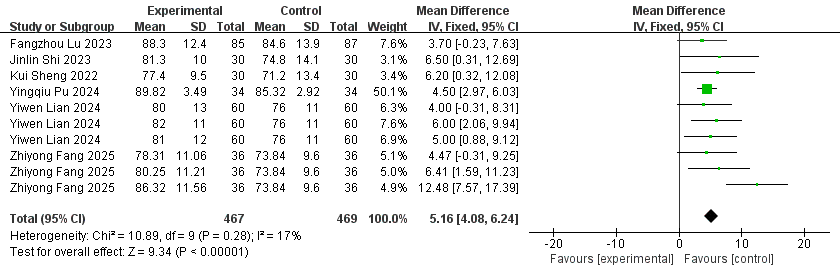


Figure S16: Comparison of mean arterial pressure at time T5 (Post-colonoscopy insertion) between groups.


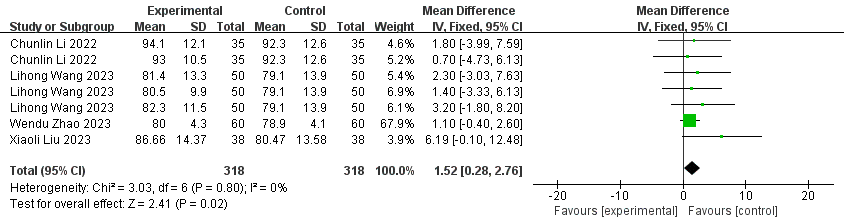


Figure S17: Comparison of mean arterial pressure T6 (Awakening) between groups.


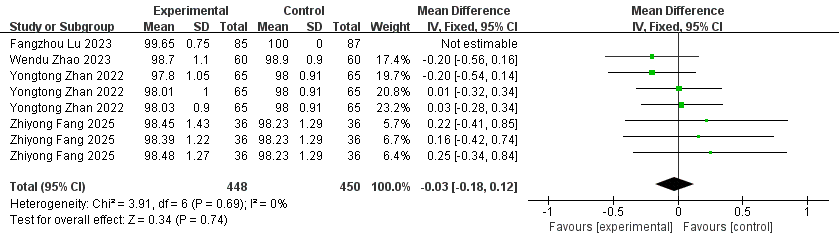


Figure S18: Comparison of peripheral capillary oxygen saturation T0 (Baseline) between groups.


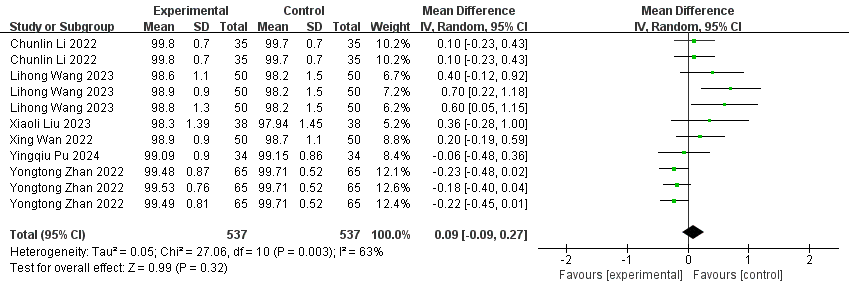


Figure S19: Comparison of peripheral capillary oxygen saturation T1 (Before induction) between groups.


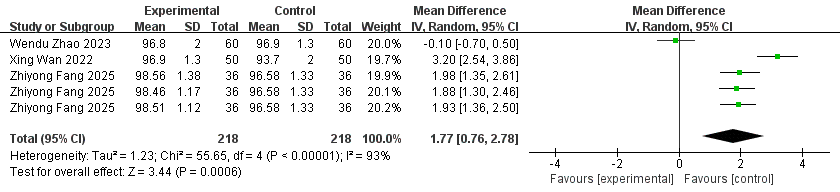


Figure S20: Comparison of peripheral capillary oxygen saturation T2 (Pre-gastroscopy insertion) between groups.


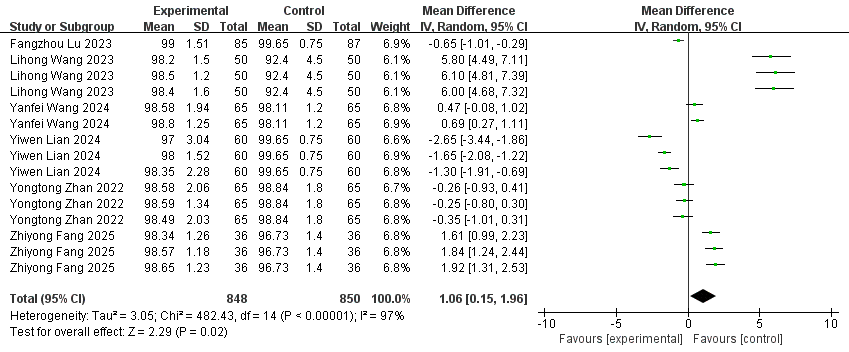


Figure S21: Comparison of peripheral capillary oxygen saturation T3 (Post-gastroscopy insertion) between groups.


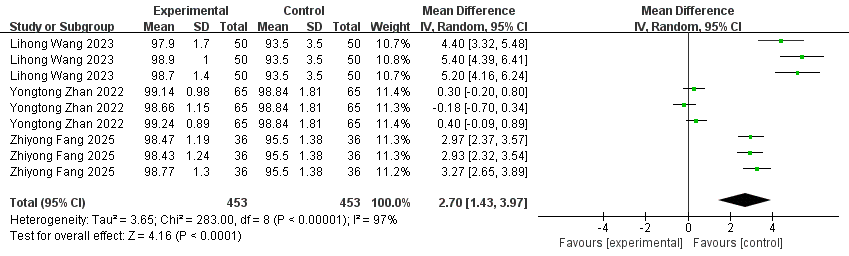


Figure S22: Comparison of peripheral capillary oxygen saturation T4 (Pre-colonoscopy insertion) between groups.


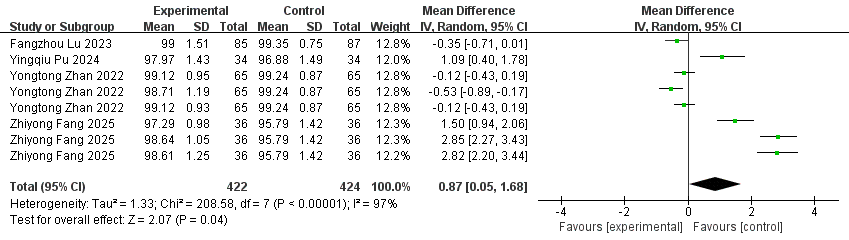


Figure S23: Comparison of peripheral capillary oxygen saturation T5 (Post-colonoscopy insertion) between groups.


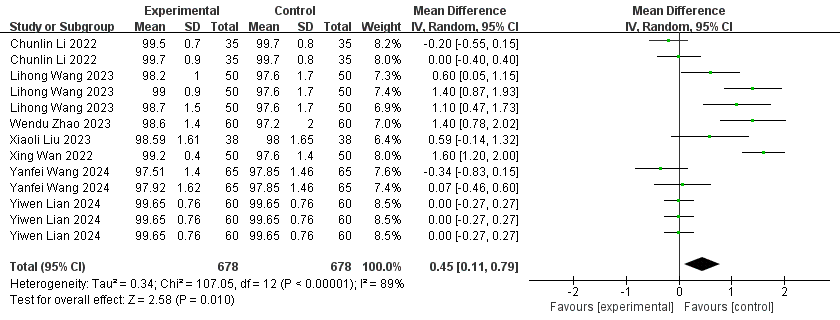


Figure S24: Comparison of peripheral capillary oxygen saturation T6 (Awakening) between groups.

**Supplementary Appendix 5.1: Time-Related Outcomes**

**
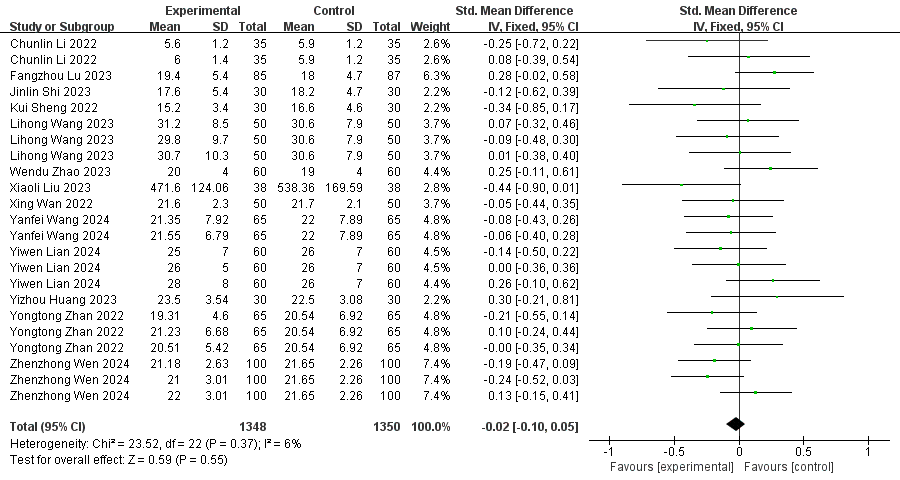
**

Figure S25: Comparison of procedure time between groups.

**
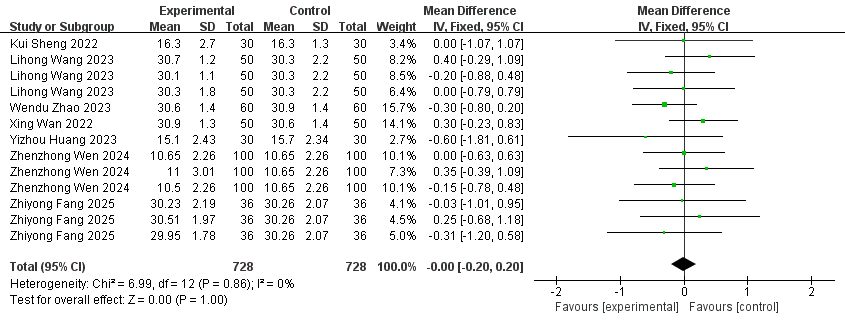
**

Figure S26: Comparison of PACU stay time/recovery time between groups.

**
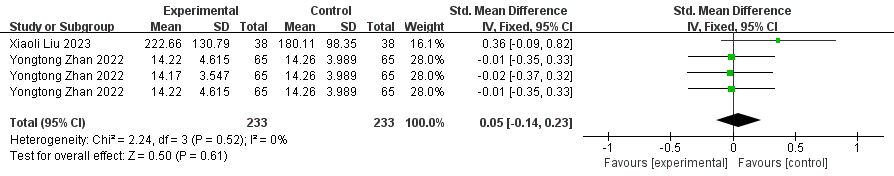
**

Figure S27: Comparison of time to regain orientation between groups.

**Supplementary Appendix 5.2: Assessment Scales**

**
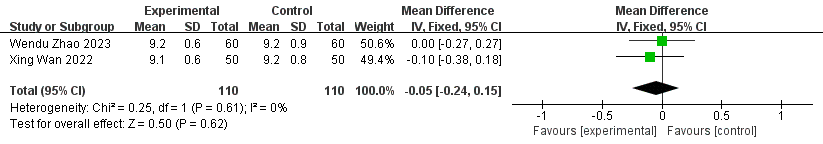
**

Figure S28: Comparison of PADSS scores between groups.

**
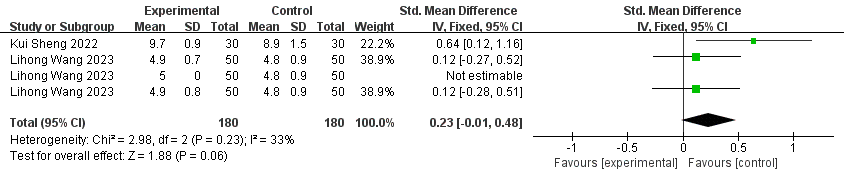
**

Figure S29: Comparison of patient satisfaction scores between groups.

**
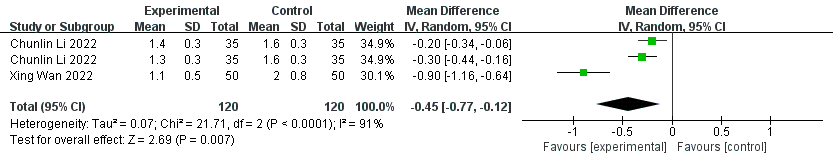
**

Figure S30: Comparison of VAS scores at awakening between groups.

**Supplementary Appendix 6: Subgroup Analysis (adverse events)**


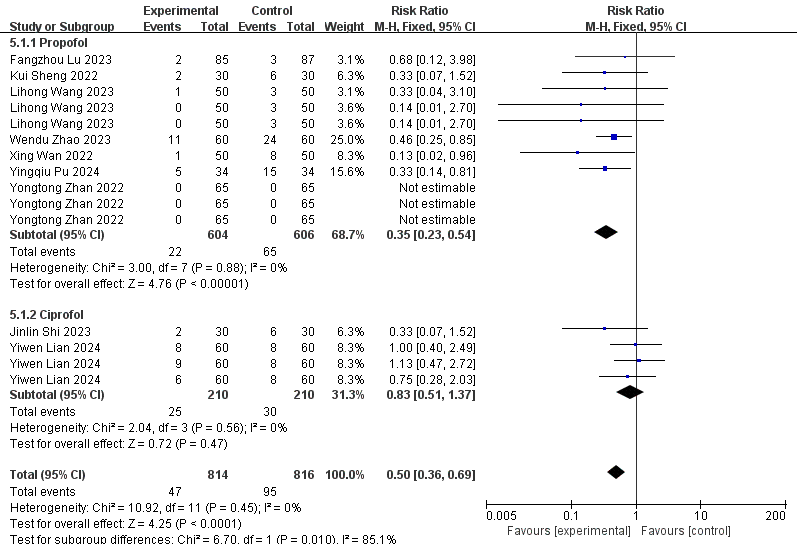


Figure S37: Comparison of the occurrence of apnea between groups (different sedatives).


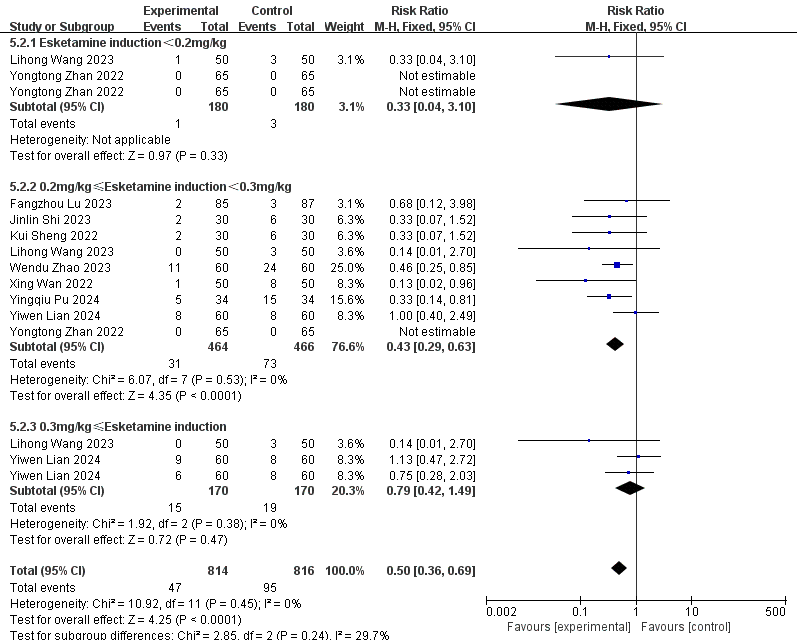


Figure S38: Comparison of the occurrence of apnea between groups (different dosages of esketamine).


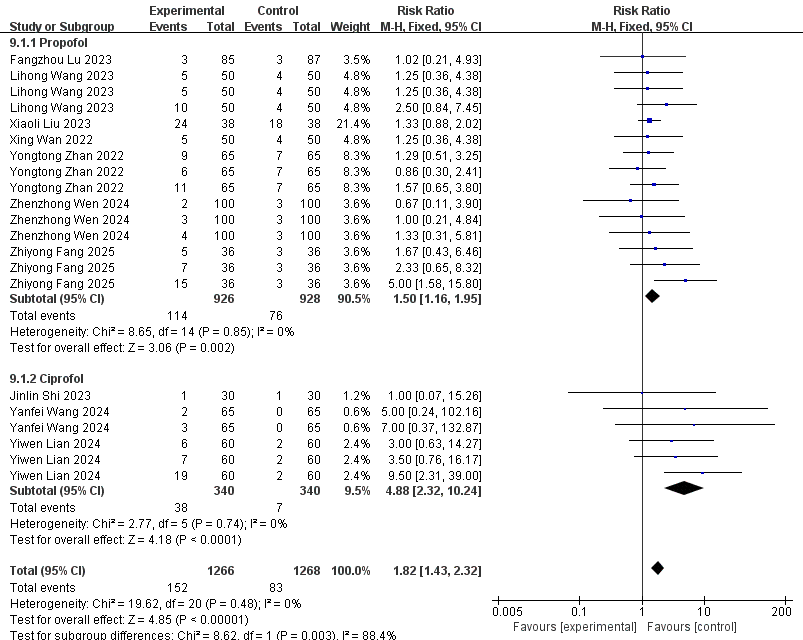


Figure S39: Comparison of the occurrence of dizziness between groups (different sedatives).


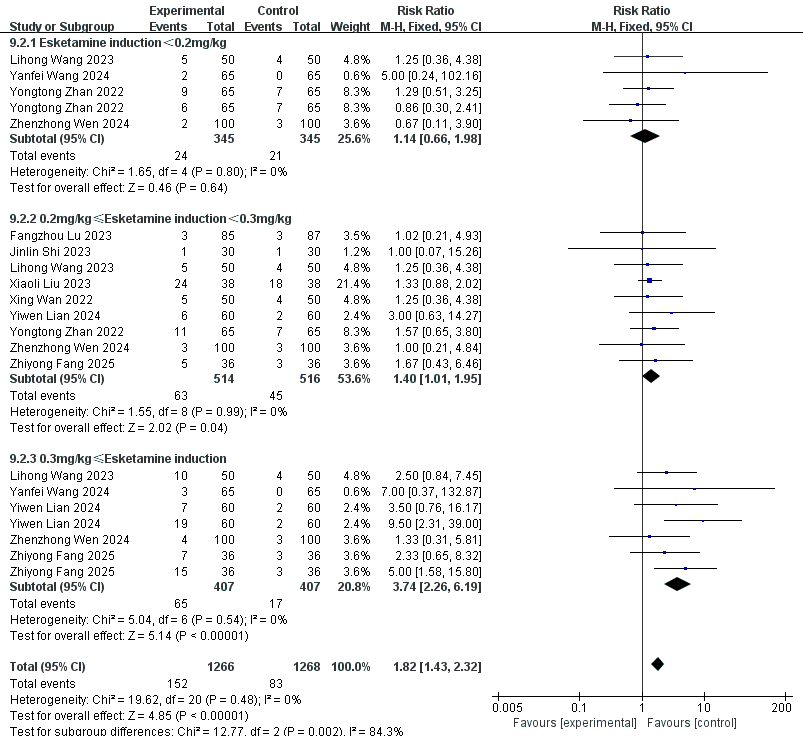


Figure S40: Comparison of the occurrence of dizziness between groups (different dosages of esketamine).


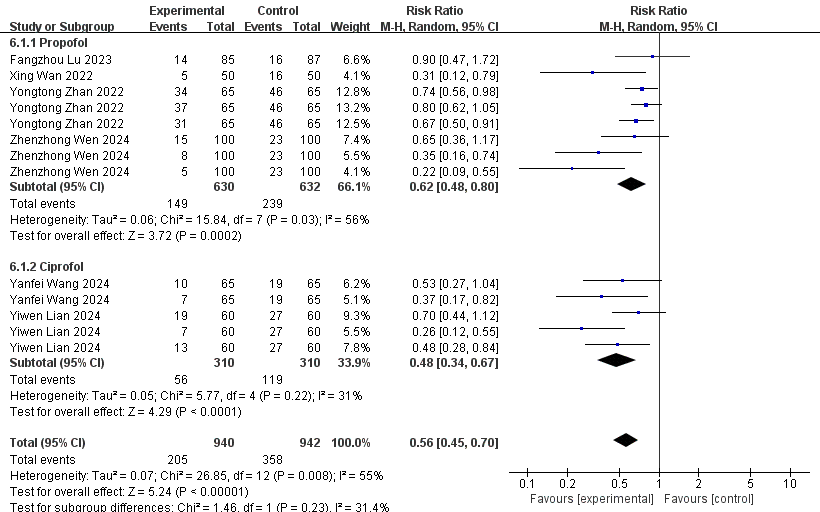


Figure S41: Comparison of the occurrence of cough between groups (different sedatives).


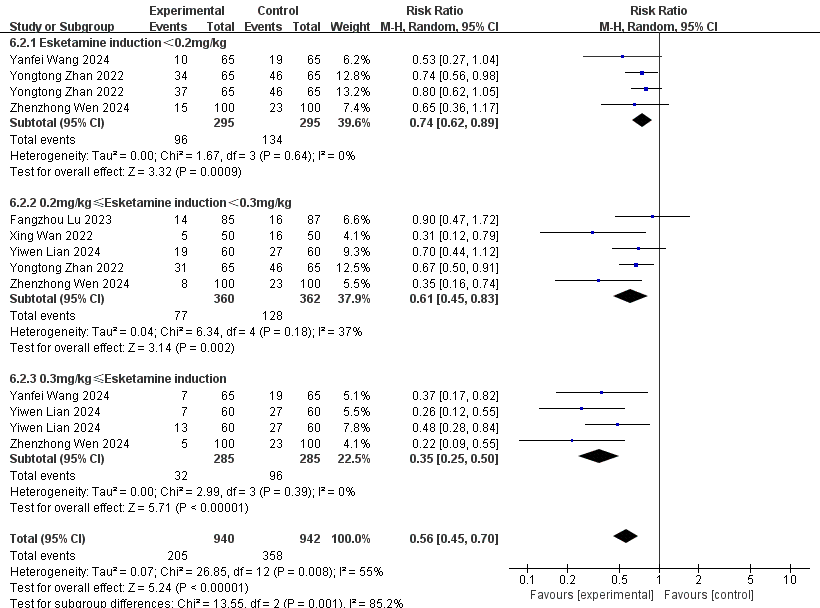


Figure S42: Comparison of the occurrence of cough between groups (different dosages of esketamine).


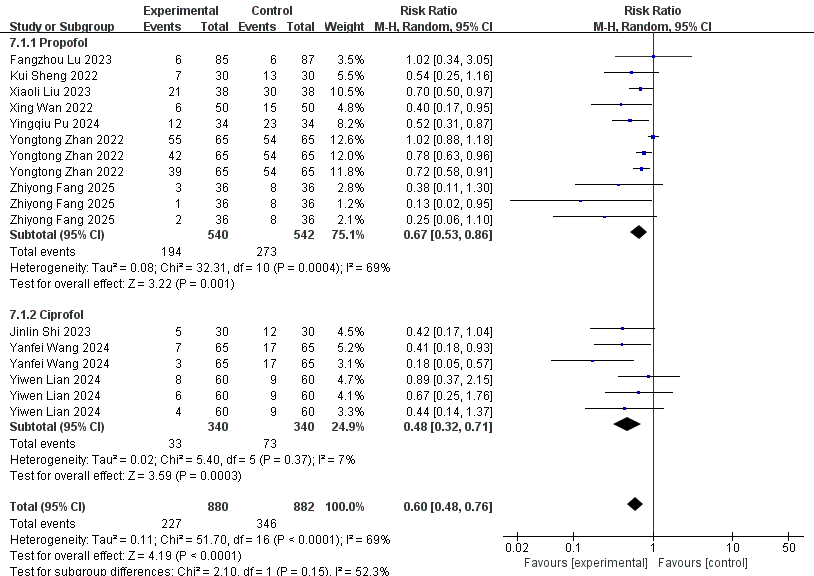


Figure S43: Comparison of the occurrence of body movement between groups (different sedatives).


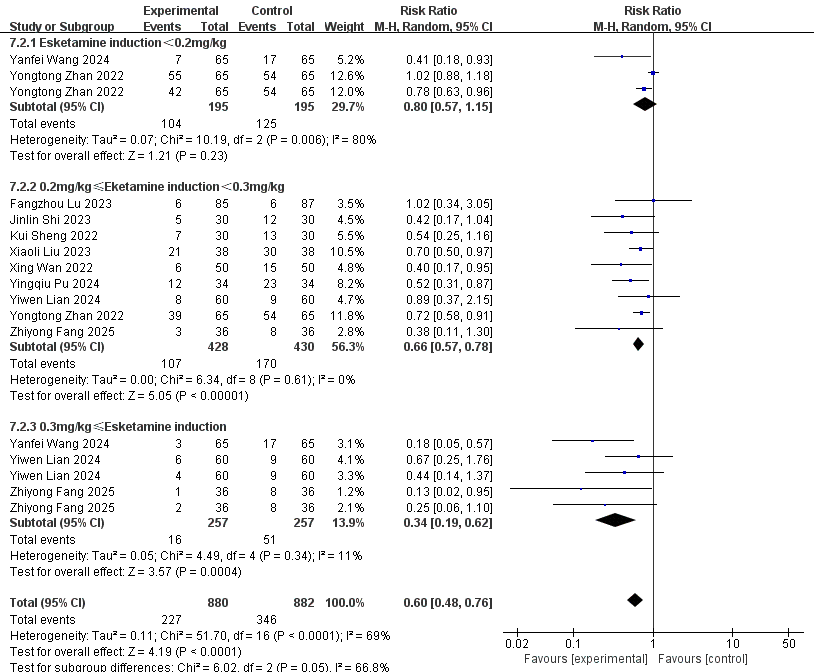


Figure S44: Comparison of the occurrence of body movement between groups (different dosages of esketamine).

**Supplementary Appendix 7.1: Funnel Plot**


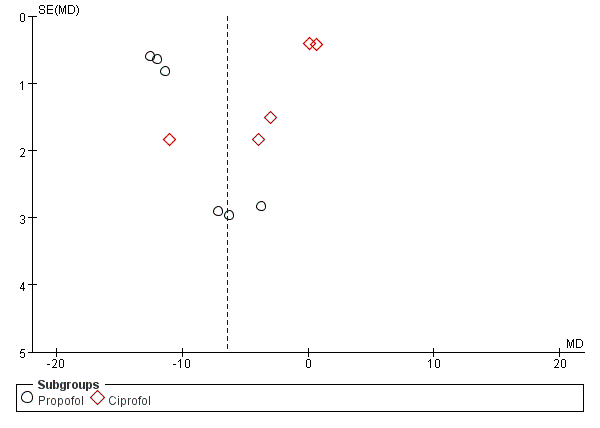


Figure S45: Anesthesia onset time (different sedatives).


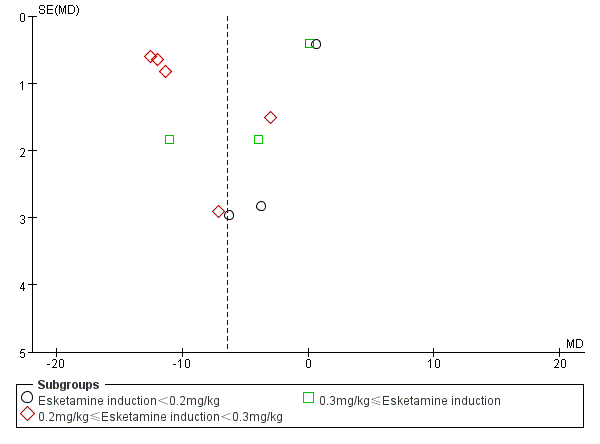


Figure S46: Anesthesia onset time (different dosages of esketamine).


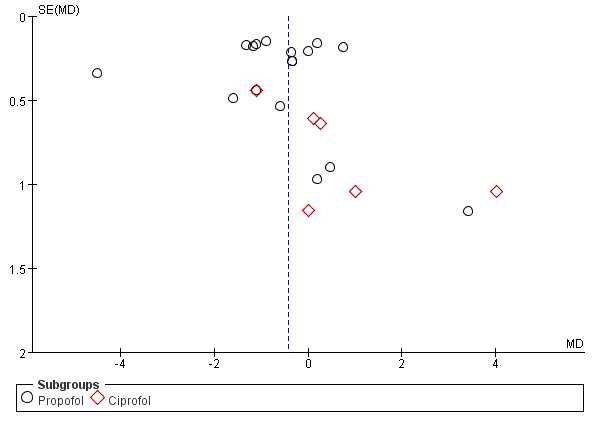


Figure S47: Recovery time (different sedatives).


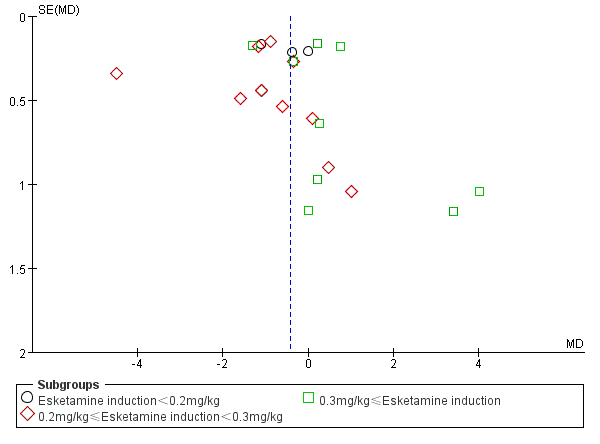


Figure S48: Recovery time (different dosages of esketamine).


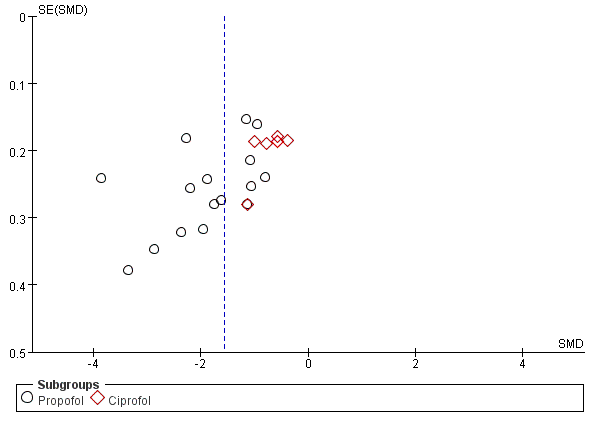


Figure S49: Cumulative sedative consumption (different sedatives).


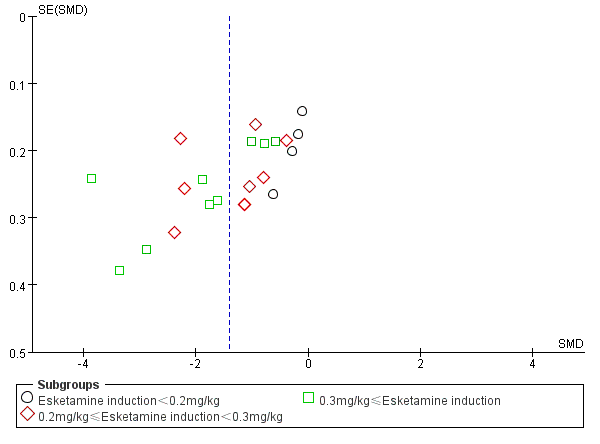


Figure S50: Cumulative sedative consumption (different dosages of esketamine).

**Supplementary Appendix 7.2: Leave-one-out Sensitivity Analyses 1**


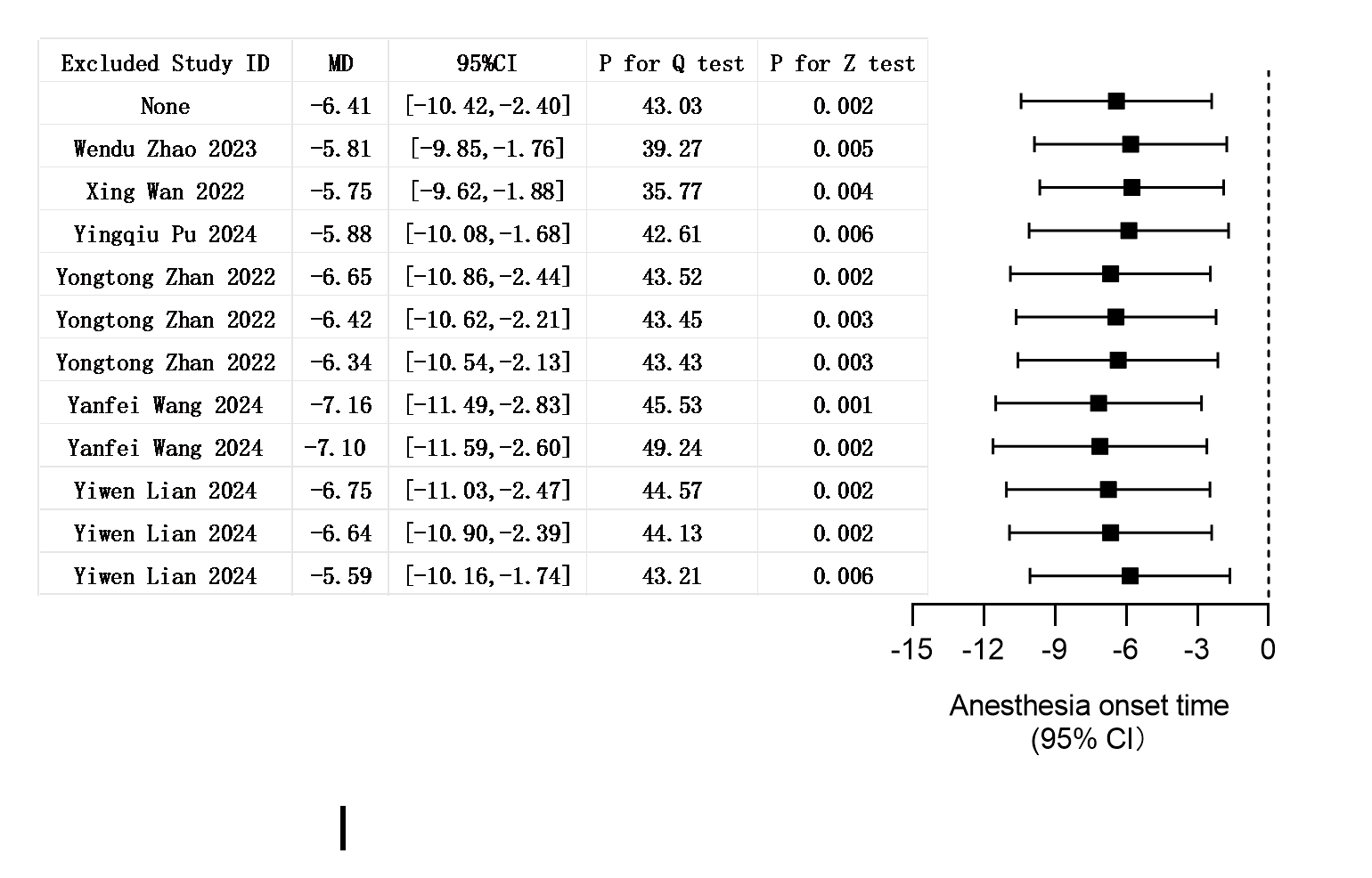


Figure S51: Anesthesia onset time.


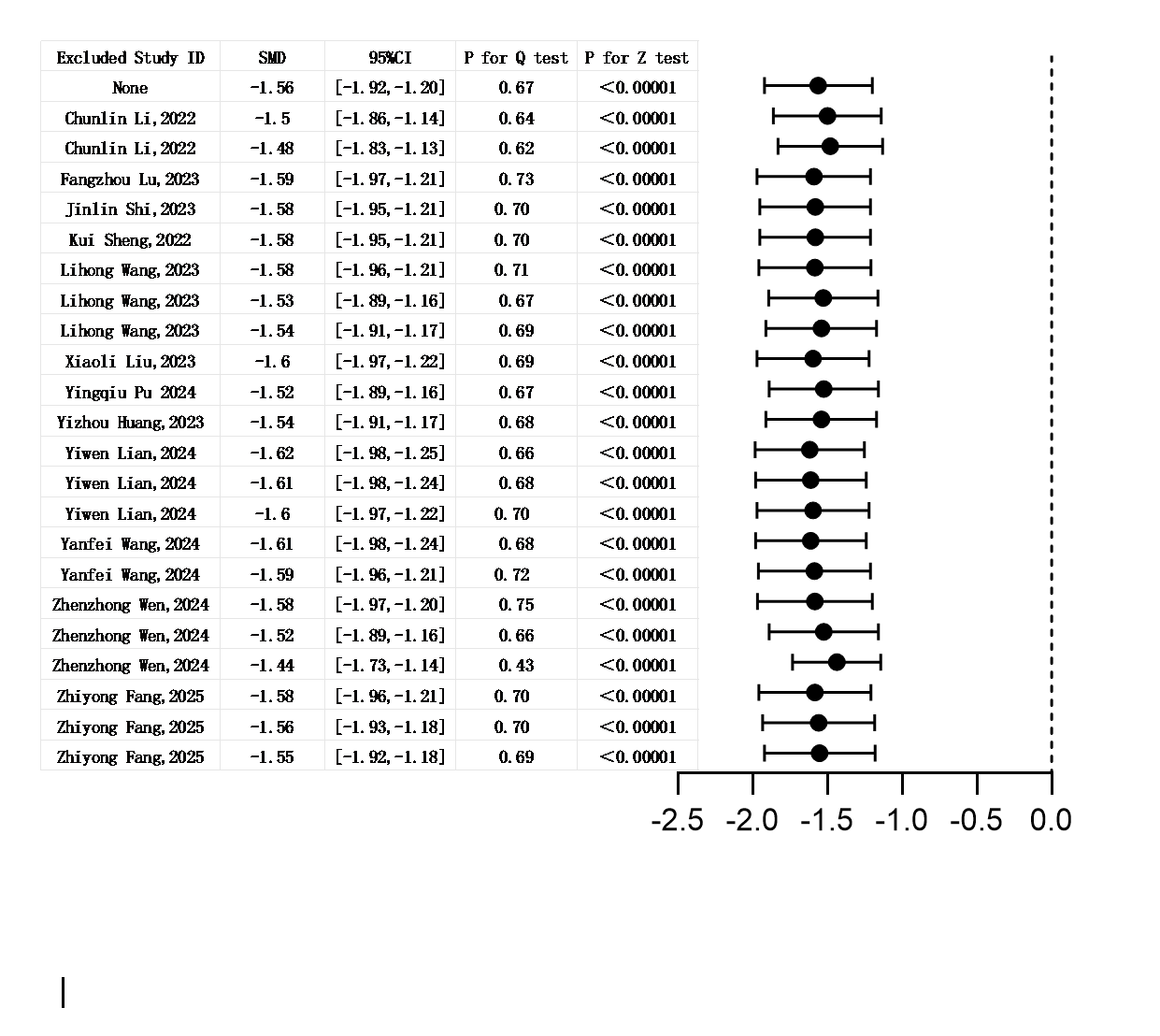


Figure S52: Cumulative sedative consumption (sum of induction and supplemental doses)

**Supplementary Appendix 7.3: Leave-one-out Sensitivity Analyses 2**


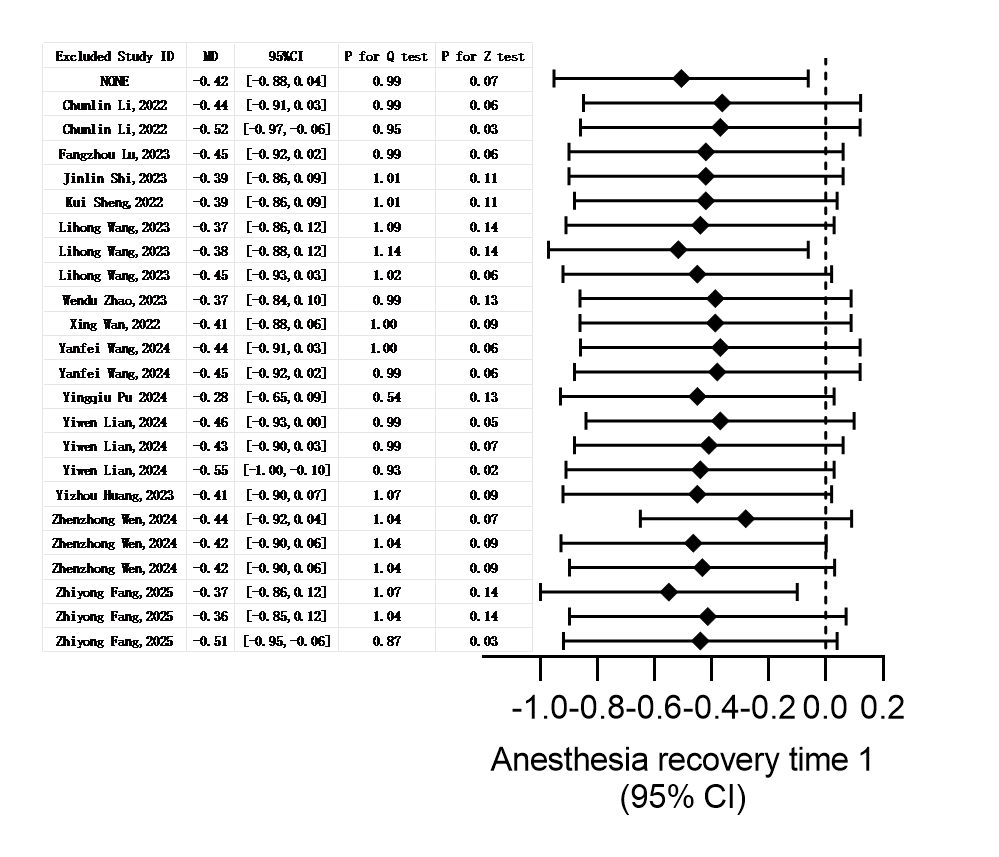


Figure S53: Anesthesia recovery time.


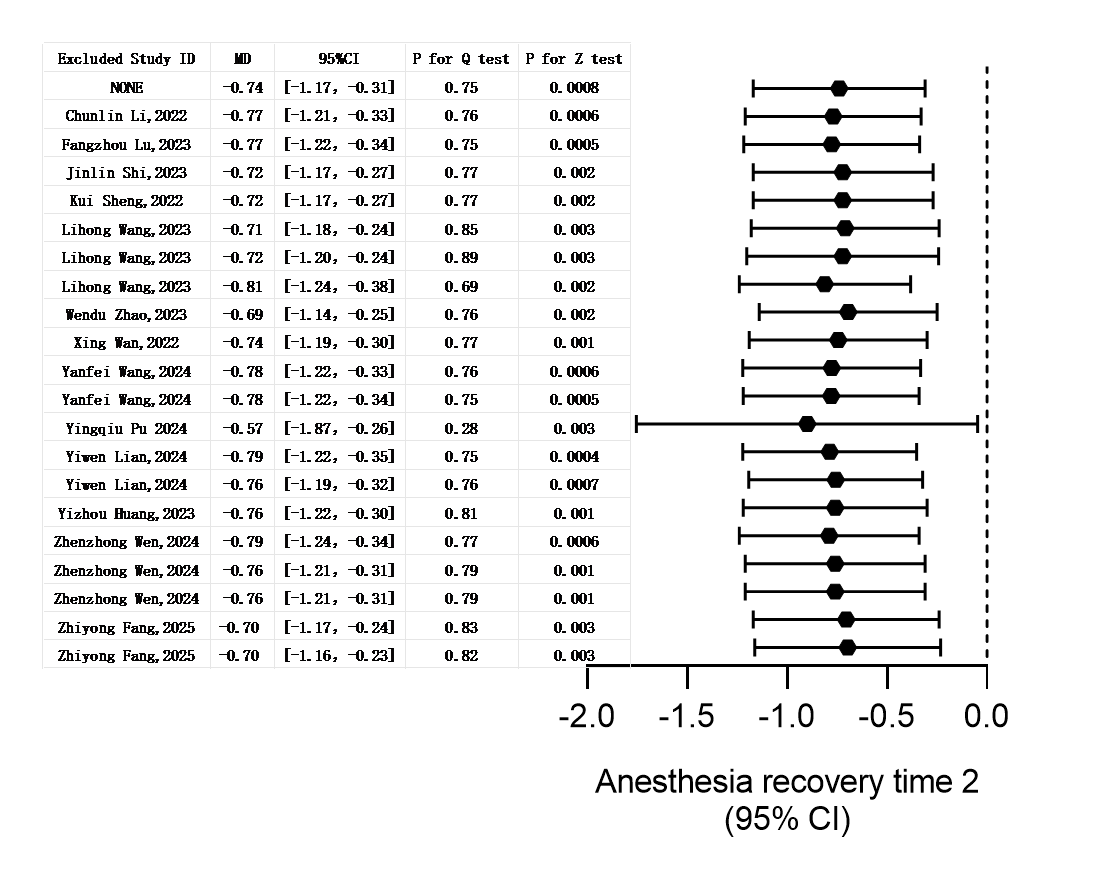


Figure S54: Anesthesia recovery time (exclusion of outliers).


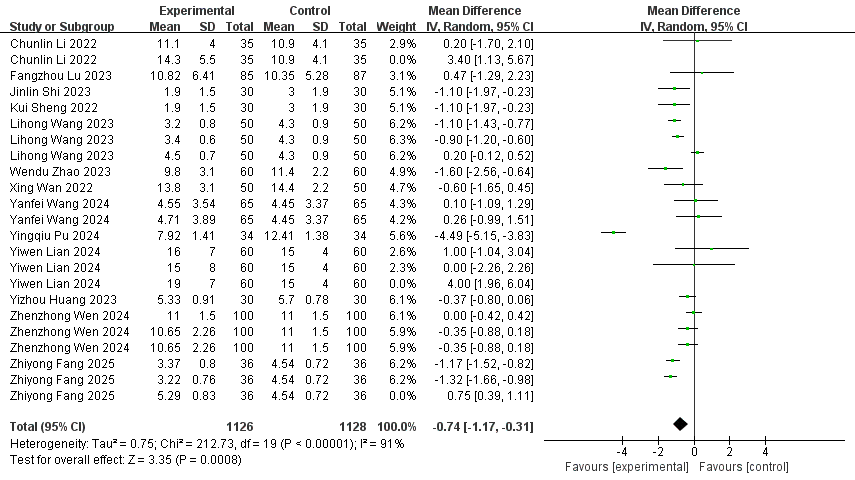


Figure S55: Comparison of anesthesia recovery time between groups (exclusion of outliers).
